# Protein-Mediated Interactions of Pancreatic Islet Cells

**DOI:** 10.1155/2013/621249

**Published:** 2013-01-08

**Authors:** Paolo Meda

**Affiliations:** Department of Cell Physiology and Metabolism, University of Geneva School of Medicine, 1 rue Michel-Servet, 1211 Geneva 4, Switzerland

## Abstract

The islets of Langerhans collectively form the endocrine pancreas, the organ that is soley responsible for insulin secretion in mammals, and which plays a prominent role in the control of circulating glucose and metabolism. Normal function of these islets implies the coordination of different types of endocrine cells, noticeably of the beta cells which produce insulin. Given that an appropriate secretion of this hormone is vital to the organism, a number of mechanisms have been selected during evolution, which now converge to coordinate beta cell functions. Among these, several mechanisms depend on different families of integral membrane proteins, which ensure direct (cadherins, N-CAM, occludin, and claudins) and paracrine communications (pannexins) between beta cells, and between these cells and the other islet cell types. Also, other proteins (integrins) provide communication of the different islet cell types with the materials that form the islet basal laminae and extracellular matrix. Here, we review what is known about these proteins and their signaling in pancreatic **β**-cells, with particular emphasis on the signaling provided by Cx36, given that this is the integral membrane protein involved in cell-to-cell communication, which has so far been mostly investigated for effects on beta cell functions.

## 1. Introduction

In vertebrates, pancreatic beta cells are the sole source of the insulin hormone [1]. The modulation of insulin secretion as a function of the changing metabolic demand and environmental conditions, specifically the levels of circulating glucose, cannot be quantitatively fulfilled by a single beta cell. Indeed, the total amount of insulin of one cell (~10 pg) will not allow for establishment and maintenance of the basal circulating levels of the hormone (~1.25 mg/L in humans). Assuming that all beta cells of the million islets which are thought to be dispersed in a human pancreas contribute to these levels, this implies that about 125 cells should simultaneously secrete in each islet. After a meal, this number should increase by about 5 to 6-fold to rapidly establish the postprandial levels of insulin, which are required to maintain normoglycemia, and be tightly regulated to ensure the peripheral oscillations of the circulating levels of the hormone, which prevent the target tissues to establish a resistance to the hormone [2–4]. Eventually, the mechanism(s) controlling these surge and oscillations should also be able to synchronously turn off the secreting cells, in order to avoid dangerous hypoglycemia, once insulin has launched its anabolic effects. Thus, insulin secretion is a multicellular process, which implies the coordinated functioning of many pancreatic islets, and many beta cells within each of these functional subunits of the endocrine pancreas. 

With evolution, many mechanisms have converged to ensure this coordination, which ensures a proper secretion of insulin [5–9]. These mechanisms include several forms of indirect cell-to-cell communication, which use extracellular molecules that simultaneously signal multiple cells, including those mediated by short- and long-range actions of multiple neurotransmitters and hormones, as well as the mechanisms that affect beta cells by several other regulatory ions and low molecular weight second messengers, purinergic signals, and short lived gases. Given that islets are separated from each other by the exocrine acini, as well as by the connective and vascular tissues of pancreas, this set of mechanisms, and notably that mediated by neurotransmitters, is believed not to be dispensable for the islet-to-islet synchronization. This set of mechanisms also controls the function of beta cells within each islet, ensuring a fine tuning of the rapidity, amplitude, rhythmicity, decrease, and duration of the insulin release. This is indicated by the experimental perturbation of these parameters *in vitro*, that is, under conditions which alter, if they do not abolish, the intercellular flux of signaling molecules and, hence, the indirect mechanisms of indirect cell-to-cell communication. The regulatory molecules are presumably generated within each islet, inasmuch as isolated islets of Langerhans retain *in vitro* a close to normal ability to modulate insulin secretion and biosynthesis in response to glucose. 

Remarkably, however, the same *in vitro* conditions do not abolish a major physiological feature of pancreatic beta cells, which is observed in no other vertebrate cell types, that is, their ability to exquisitively sense minute changes in the levels of circulating glucose, and to regulate accordingly the level of insulin secretion. In contrast, this cell-specific feature is rapidly lost once beta cells loose the contacts which they natively establish with each other, and other types of endocrine cells, within the pancreatic islets. Since a partial recovery of this loss is acutely observed after cell reaggregation [5–9], at least some of the many surface proteins which become functionally activated upon beta cell contact appear obligatory for proper insulin secretion. 

Like all other types of epithelial cells, beta cells closely adhere to their neighbors by a variety of cell surface proteins [5–9], many of which are members of multigene families. These proteins selectively interact within restricted domains of the cell membrane to form intercellular junctions, or form channels permeable to a variety of ions, metabolites, and second messengers. Some junctions establish adhesive links between adjacent cells, ensuring the structural cohesiveness of the islet, and contribute to the functional polarity of secretory cells, by establishing distinct membrane domains. Other junctions provide for anchoring of the endocrine cells to extracellular pancreas components, which presumably allows for the establishment of pathways that transduce signals within and between cells, in order to couple extracellular changes with intracellular responses. Some channels establish direct exchanges of cytosolic components between adjacent cells, which allows for the synchronization of companion beta cells. Other channels may mediate the coordination of the beta cells with the surrounding alpha cells, which produce glucagon antagonistically with insulin secretion, as well as with the other types of islet cells, including the delta cells, which produce somatostatin in parallel with insulin secretion, the PP cells, which produce pancreatic polypeptide, and the epsilon cells, which produce ghrelin. Together, this set of mechanisms of direct communication ensures the integration of these different cell types within structurally and functionally coherent pancreatic islets [5–9]. Typically, these mechanisms operate over a small distance range, due to their dependence on cell-cell or cell-extracellular material contact, and because they are ofter diffusion driven, thereby providing a potential clue as to the intriguing small size of pancreatic islets, which has been consistently selected in most animal species [10].

This paper reviews the proteins involved in these direct cell communications [8, 9], and the mechanisms whereby they ensure direct islet cell adhesion (cadherins and Ca^2+^-independent junctional molecules), anchoring to the extracellular matric (integrins), polarity (claudins and occludin), and possibly communications between beta cells and other islet cell types. Specific attention is given to Cx36, the sole connexin expressed by pancreatic beta cells, since increasing evidence points to a relevant *in vivo* role of the coupling that this protein ensures within the islets, in multiple aspects of beta cell functions. 

## 2. Why Cell-to-Cell Interactions?

A first multi-cellular organism is believed to have formed between cyanobacteria some 3.5 billion years ago, relatively soon after the earth crust solidified [11]. Since, this event repeated itself a number of times [12–20] till about 800 million years ago, when it initiated the development of the larger algae, fungi, plants, and animals we now know [13–16, 21, 22]. This development was accompanied by increased genomic diversity, presumably as a result of the recruitment by multicellular organisms of genes from several unicellular ancestors [18, 19]. This recruitment, together with a series of spontaneous genetic mutations and environmental changes, is the likely cause of the increased size of the newly formed multicellular organism [12, 17]. In turn, this change lead to cell diversity, due to the necessity to sustain the larger body with novel metabolic and structural adaptations [21]. Thus, multiple cell types emerged [16, 21], imposing to the multicellular organism to transform from a mere aggregate of independent cells into a community of interacting cells. The new organisms presumably were selectively advantaged by these changes, since phylogeny shows a trend towards increased organism complexity [18, 19]. In turn, multicellularity provided control over the environment. This facilitated the independent determination of the intracellular medium and the constitution of a stable *milieu interieur, *which protected the multicellular organisms from environmental alterations [16]. 

## 3. How Cell-to-Cell Interactions?

This phenomenal and explosive evolution dependeds on the parallel development of a communication array for cross-talk between the various types of cells that strived to live together. Via such an array, adjacent and distant cells were forced to coordinate their function with that of other cells, to optimize their adaptation to the environment [23, 24]. Likely, this signaling array was initiated by the diffusion across the cell membrane of small signaling molecules [12, 25–32]. Since, selection has largely diversified this primordial mechanism, resulting in a complex array of cross-talking and to some extent overlapping cell communication mechanisms, which use different structures and signal molecules [8, 9]. These mechanisms are referred to as “indirect” or “direct” cell-to-cell communication mechanisms. Indirect cell communication does not require cell contact, being mediated by the extracellular flux of molecules that simultaneously signal multiple cells. A widespread modality of such a mechanism, is the diffusion in the extracellular spaces of hormones and neurotransmitters, which are simultaneously sensed by cells equipped with cognate receptors [28, 29, 31, 32]. This system ensures a highly specific signaling between both distant (hormonal and neural communication) and nearby cells (paracrine communication), sometimes even affecting the very same cell that generated the signal (autocrine feed-back loop). Another widespread form of indirect cell-to-cell communication is provided by the diffusion in the extracellular spaces of ions and molecules that enter cells either by free diffusion through the lipid membrane bilayers or by way of specific transporters/channels [33, 34]. A recently recognized variant of this modality is the release through membrane pannexin channels of cytosolic signal molecules which enter the extracellular fluid and then interact with specific receptors in close-by cells. Cell-to-cell coordination is then achieved by the simultaneous up- or downmodulation of specific metabolic and effectors pathways in several cells. A further modality of “indirect” cell-to-cell communication is the dispersion of the extracellular cell-to-cell signal by components of the extracellular matrix, to which multiple cells may simultaneously and dynamically attach by way of specific integrin proteins, that mediate both in-out and out-in signaling [8, 9]. 

Possibly because multicellularity initially developed in large aqueous media, most cell types also developed ways to communicate in a “direct” way, that is, by mechanisms which require their physical contact at the level of the cell membrane [8, 9]. A widespread modality of such direct cell-to-cell communications is via a large variety of cell adhesion glycoproteins, referred to as cell adhesion molecules (CAMs), which establish and maintain cell cohesion, while transmitting both intra- and extracellular information to adjacent cells [8, 9]. Direct cell-to-cell communication is also mediated by connexins [35–39], the nonglycosylated proteins that oligomerize to form hydrophilic cell-to-cell channel, which functionally joins two cytoplasms, thus allowing for bidirectional exchanges of a variety of cytosolic molecules [35–39]. 

Probably because most of these indirect and direct cell communication mechanisms operate in a relatively short distance range, they were not, alone, sufficient to cope with the increasing size and complexification of multicellular systems, which progressively included increasing numbers of cells, differentiating in multiple directions [15, 39]. Thus, the nervous and endocrine systems emerged to carry the signal molecules to both close-by and distant target cells [12, 26, 27, 40, 41]. The use of similar signal molecules and signal molecule-receptor interactions all along the phylogenetic tree suggests that the two systems evolved from a common ancestor [28–32, 40, 42–46], presumably by random modification of some common metabolite [27, 47]. Since, comparative biology has shown little evolutionary changes in the type of hormones produced by different organisms [27, 40, 42], but a large diversification of the types of cells targeted by the signaling molecules, and, to a lesser extent, of the pathways which are controlled by hormones and neurotransmitters in these cells [27, 40, 48]. As a result, a same signaling molecule may have markedly different effects in different species [27]. With the advent of large size vertebrates, the requirement for hormones increased, forcing the development of multicellular glands, where many cells would concentrate to produce one or several signaling molecules. Again, there has been but a modest phylogenetic adaptation of these glands, even when their spatial architecture changed, indicating some need for convergent evolution [25, 27, 49, 50].

## 4. Hormone Secretion Is a Multicellular Event

The phylogeny of endocrine glands recapitulates several steps of the evolution of multicellularity, notably the essential need for intercellular communication to ensure a coordinated function of the endocrine secretory cells that are vital for mammal survival. Thus, secretion of vital hormones is a multicellular process, given that no individual cell can produce, at any given time, the large amount of hormones that is required to ensure their vascular transport across a large organism, and to regulate the peripheral target organs. Hence, endocrine cells making a specific hormone should synchronize their activity with companion cells, and coordinate this activity with that of cells producing agonistic or antagonistic hormones, which may not function synchronously and at the same rate [51–55]. Since most hormonal secretions undergo regular fluctuations, as a function of circadian and other rhythms, and are affected by changes of the *milieu interieur*, the extracellular *milieu* of the organism and the environment, this coordination should be continuously adapted, on a moment-to-moment scale, to provide a hormone output commensurate with the needs of the organism. 

Endocrines meet these requirements, by using in parallel, though not necessarily simultaneously, all the indirect and direct forms of cell-to-cell communication mentioned above [51–53, 55–57], in patterns which differ from gland to gland. This organization results in a complex network of different regulatory systems, which largely overlap and, at times, interact with other pathways to promote or decrease their influence. Most likely, this partial redundancy was pressure-selected by the vital necessity of some of the hormones, like insulin or corticosteroids. Providing the cells making these hormones with many regulatory systems ensures that the biosynthesis, storage, and release of these molecules are maintained within a life sustainable range under most conditions, including when individual regulatory mechanism may have been lost. Distinct communication mechanisms, each resulting in quantitatively and qualitatively different effects, also provide endocrine cells with a much more sensitive and graded way to control their functioning, in both temporal and spatial terms, than possible using a single, on/off type of regulation. 

## 5. Cell Interactions in the Endocrine Pancreas 

### 5.1. What Is the Endocrine Pancreas?

In mammals, the endocrine pancreas is collectively made by a multitude of islets of Langerhans which are dispersed within the pancreas. Each islet is a round/ovoid mass, 50–600 *μ*m in diameter, which comprises ~50–3000 cells, of which beta cells represent about 60% in humans. The remaining cells are glucagon-producing alpha cells, somatostatin-producing delta cells, pancreatic polypeptide-producing cells, and ghrelin-producing epsilon cells. The islets also comprise abundant endothelial cells, as well as some fibroblasts, lymphocytes, and macrophages [58]. This complex organization, and the vital necessity of these microorgans contrast with their actual minute amounts. In humans, the estimated number of pancreatic islets is one million per pancreas, under healthy, control conditions, which means that our survival is dependent on about one billion beta cells. Still, these cells altogether only form 1-2% of the volume of a control, adult human pancreas, and represents no more than 1 g wet weight tissue [58]. 

### 5.2. Interactions between Pancreatic Islets

Under resting conditions, the three main islet hormones, insulin, somatostatin and glucagon, are all released in an oscillatory manner [2–4, 59–62], which is synchronous for the two former hormones and antisynchronous for the latter [63]. After a meal, the release of insulin and somatostatin increases sharply, whereas that of glucagon decreases. Conversely, during fasting, insulin and somatostatin are downregulated, whereas the release of glucagon is increased. This regulation implies a coordination between both multiple islets, and between their different cell types, since a stochastic function of individual islets is unlikely to ensure both the acute and important post-prandial surges in the hormone levels, their regular, cyclic fluctuations over time, and their rapid off response when the nutrient stimulation ceases. Given that islets are separated from each other by basal laminae, connective tissue, and the acini of the exocrine pancreas, their coordination depends on a variety of hormonal and neural controls. Thus, insulin secretion is experimentally stimulated by gastric inhibitory peptide, thyrotropin-releasing hormone, glucagon-like peptide 1, *β*-adrenergic agonists, acetylcholine, cholecystokinin, gastrin-releasing peptide, and vasoactive intestinal peptide [64–67]. Conversely, inhibition is induced by corticotrophin-releasing factor, peptide YY, atrial natriuretic peptide, pancreastatin, *α*_2_-adrenergic agonists, galanin, neuropeptide 1, and somatostatin [64–67]. *In vivo*, the insulin secretion of islets, which is initiated by circulating nutrients, mostly glucose, is essentially modulated by gut hormones, specifically gastric inhibitory peptide, glucagon-like peptide 1, cholecystokinin, and somatostatin [64–67]. *In vitro*, pulsatility of insulin secretion is preserved in the absence of hormonal circulation [2, 3, 66–68], further indicating that some intrapancreatic nervous system helps coordinating the individual islets. Since insulin pulses are altered by tetrodotoxin, but not by drugs blocking cholinergic and adrenergic receptors, this islet coordination presumably involves connections between islets, mediated by postganglionic fibers of autonomous ganglia [64]. These ganglia, which serve both as pancreatic pacemakers and as integration centers [66], receive adrenergic, cholinergic, and peptidergic inputs from both the central and the autonomous nervous systems [66].

### 5.3. Interactions within Pancreatic Islets

Within each pancreatic islet, endocrine and vascular cells communicate in a variety of ways which presumably include all the indirect and direct mechanisms listed above. Thus, it has long been known that the insulin-producing beta cells regulate their functions through interactions with the same hormones, neuromediators, and other signal discussed in the previous paragraph for islet communication. These signal molecules reach the pancreas from outside or are generated within the islet itself [6, 57, 64, 69, 70]. However, given that insulin secretion is still a regulated event under *in vitro* conditions which abolish the native blood supply and perturb both the innervation and the flux of extracellular fluid, other communication mechanisms must contribute to beta cell control. The finding that insulin secretion is altered after dispersion of islet cells and rapidly improves after their reaggregation or their interaction with components of the extracellular matrix [71–78], further suggests that these mechanisms depend on the establishment and maintenance of proper cell-to-cell and cell-to-matrix contacts. 

#### 5.3.1. Indirect Interactions 

*Neurotransmitter and Hormone Mediated*

 Individual islets of Langerhans respond to most of the neuromediators and hormones which affect the endocrine secretion of the intact pancreas, including the four main islet hormones [69, 70, 79]. Thus, insulin inhibits glucagon secretion, and possibly also the secretion of somatostatin, pancreatic polypeptide, and insulin itself; glucagon stimulates insulin and somatostatin secretion; pancreatic polypeptide inhibits somatostatin secretion; and somatostatin inhibits the release of all islet hormones [69, 70, 79]. Some of these effects can be prevented by antibodies to specific hormones, suggesting that they may well be elicited *in vivo* after release of endogenous islet products [69, 70, 79]. Since islet cells are influenced by hormone levels much lower than those found in the venous effluent of the pancreas, it is difficult to conceive that any paracrine effect may occur by diffusion of hormones from the producing cell to nearby targets, through a continuous extracellular space. If this was the case, it is likely that the islet cell receptors may be downregulated by the sustained exposure to high hormone concentrations [69, 70, 79]. Thus, it is likely that islet hormones are channeled to specific membrane domains, either by restricted diffusion in the extracellular islet space or by vectorial transport via the islet microcirculation [64, 66, 69, 70, 79]. Microvessels originate from afferent arterioles in the center of the islets which, in the rodents that were used in these experiments, predominantly comprise insulin-producing beta cells and direct blood flow to the islet periphery [80], a region comprising beta, alpha, delta, PP, and epsilon cells [58, 81]. Comparison of arterial and venous infusions, in the presence and absence of hormone-neutralizing antibodies, suggests that blood flow reaches beta cells first, then alpha cells, and finally delta cells [79, 80]. According to this arrangement, high concentrations of insulin should almost continuously bath the peripheral islet mantle, whereas glucagon and somatostatin would have little chance of reaching most beta cells without first entering systemic circulation. However, whether and how all islet hormones actually have any intraislet effect *in vivo* is still the subject of substantial debate, since we still do not know the actual concentration of hormones in the interstitial islet fluid, the flux and direction of this fluid, and the distribution of islet cell receptors. Recent evidence has certainly provided evidence that at least insulin signaling is significant for islet function. Thus, invalidation of the gene coding for insulin receptors of pancreatic beta cells leads to mice featuring a selective loss of insulin secretion in response to glucose, which was sufficient to impair glucose tolerance [80]. Autonomic ganglia have not been observed within isolated islets [64], indicating that basal and stimulated insulin secretion may be sustained in the absence of innervation. Nevertheless, interruption of extraislet neural inputs from both parasympathetic and sympathetic fibers modifies islet function, as revealed by an increased frequency in the oscillations of insulin secretion, in rodents [2]. The rapid oscillations observed with individual, isolated islets are probably driven by periodic fluctuations in the levels of intracellular free Ca^2+^ and/or in the glycolytic activity of beta cells [61]. *In vivo*, these oscillations may be masked by the longer cycle pulses that result from the neural coordination of multiple islets [4, 63, 66]. Whether these considerations apply to human islets, in which the beta cell and alpha cell compartments are not as regionally distinct as in rodents [58, 81], and whose innervation also appears much sparser than in rodents [82, 83], remain to be shown.

*Ion Mediated*

 Numerous other, nonhormonal or neural signals which flow through the extracellular spaces of the pancreatic islets may help coordinating beta cell behaviour. For example, it has been noted that electrophysiological characteristics are quite variable at the level of single beta cells and become more uniform and stable when these cells aggregate into clusters [61, 81, 84]. Within intact islets, virtually all beta cells show a high degree of electrical synchronization during both silent and burst periods of electrical activity [61, 81, 84–87], implying an almost immediate intercellular coordination of the levels of current-carrying ions. This coordination has been verified for Ca^2+^, whose oscillatory levels appear to be determined collectively by groups of synchronized beta cells rather than by individual *β*-cells [61] and may rapidly equilibrate across whole islets [88, 89]. We now know that this equilibration is mostly dependent on Cx36 channels [59, 62], even though other regulatory mechanisms may modulate this essential function [3, 4]. Thus, the occurrence in the extracellular islet fluid of K^+^ waves, which regularly precede the bursts of electrical activity [90, 91], may provide a rapid and efficient way for coordinating the depolarization of the beta cell membranes, as well as the subsequent Ca^2+^ oscillations and insulin release in distant beta cells [6, 54, 57]. Still other islet cell interactions may involve signaling by either cytokines or NO [91–94].

*Integrin Mediated*

**Figure 1:**
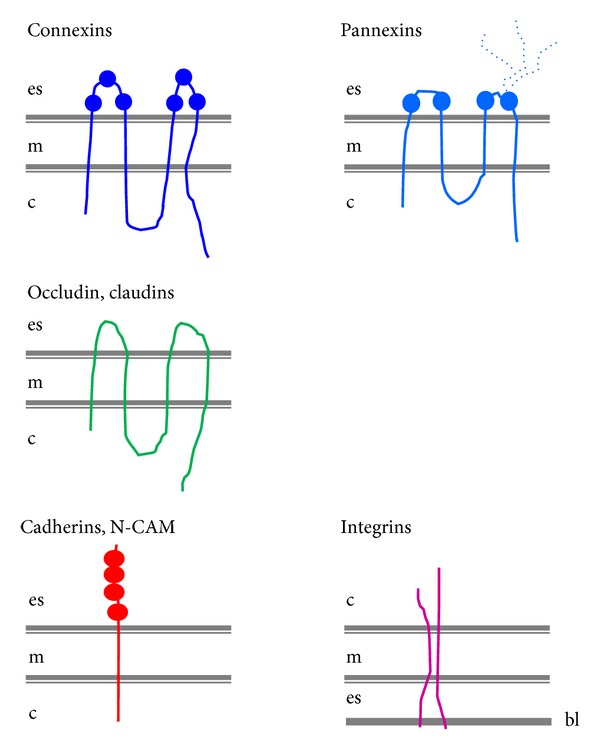
Schematic representation of the membrane proteins involved in *β*-cell communication. Pancreatic islet cells express molecules for cell-to-cell communication, including tetraspan connexins, pannexins, occludins, and claudins, and single span cadherins, and N-CAM. Pancreatic islet cells also express molecules for cell-to-extracellular matrix communication, notably integrins. The spherical symbols on the extracellular loops of connexins and pannexins indicate the presence of highly conserved cysteine residues. The branched, dotted line on the second loop of pannexins indicates the site of glycosylation.

**Figure 2:**
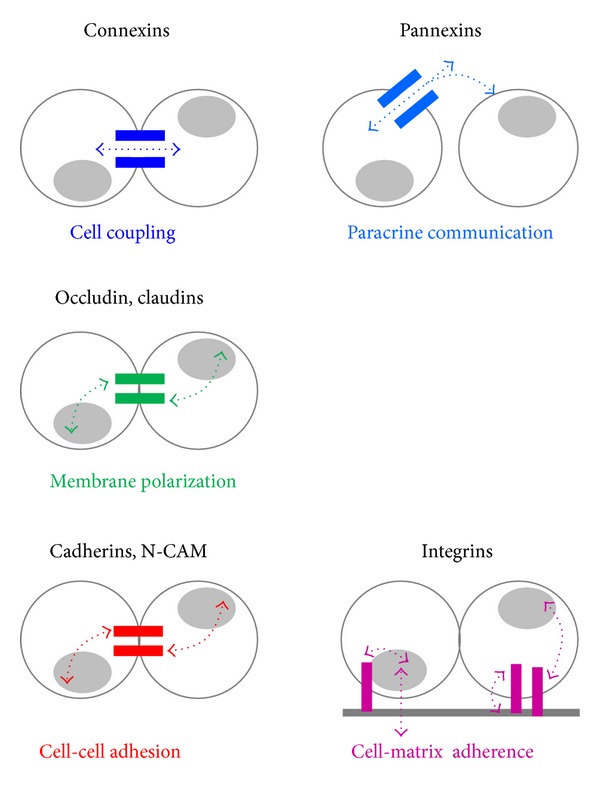
Different membrane proteins account for different modalities of *β*-cell communication. Connexins mediate the direct cell-to-cell transfer of cytosolic molecules (dotted, double arrow head) between adjacent cells, an event referred to as cell-to-cell coupling. Pannexins mediate the exchange of molecules (dotted) between the cytosol and the extracellular space of cells. Some of the cytosolic molecules which exit cells through pannexin channels could ensure paracrine communication between close-by cells. Occludin and claudins provide for the sealing of small domains of the cell membrane and of portions of the islet extracellular spaces, hence establishing the polarity of *β*-cells. Cadherins and N-CAM ensure the adhesion between islet cells in contact. Integrins mediate the adherence of islet cells to the extracellular basal lamina (grey band) and matrix. Claudins, cadherins, N-CAM, and integrins also provide for the in-out and out-in signaling of *β*-cells (curved, double arrow heads) notably with regard to gene expression.

Numerous cell surface proteins have been identified as receptors for molecules of the extracellular matrix (ECM). Almost all of these proteins belong to the integrin family, which are receptors composed of an *α* (120–180-kDa) and a *β* subunit (90–110 kDa), noncovalently bound (Figure 1). Each subunit has an extracellular domain, a single transmembrane region, and a short cytoplasmic domain associated, via a variety of cytosolic protein partners, with actin microfilaments [95]. To date, 18 *α* and 8 *β* subunits have been identified in vertebrates, which assemble into at least 24 distinct integrin isoforms. Typically, different integrins serve as receptors for different components of the ECM (Figure 2). However, different integrins may also recognize the same ligand and, conversely, integrins composed of a same subunit may show different ligand binding specificities [95]. Integrins initiate the adhesion of cells to the substratum, on which they grow (basal lamina and ECM for epithelial cells), and organize their cytoskeleton, leading to changes in cell shape, polarity, and distribution of cellular organelles. They further activate a large variety of signal transduction events that modulate many aspects of cell behaviour, including proliferation, survival/apoptosis, shape, polarity, motility, gene expression, and differentiation [96–99]. Integrins are not endowed with enzymatic activity, but are associated with a broad variety of signaling and/or adaptor proteins involved in different signal transduction pathways. Frequently, integrin-mediated adhesion and/or clustering of integrins leads to enhanced activation of a cytoplasmic integrin-associated protein kinase, known as focal adhesion kinase (FAK) [100, 101]. A multitude of signaling molecules, including PI3K, protein kinase B (PKB/Akt), and the MAP kinase ERK, are regulated by integrin-mediated adhesion [100–102].

**Figure 3:**
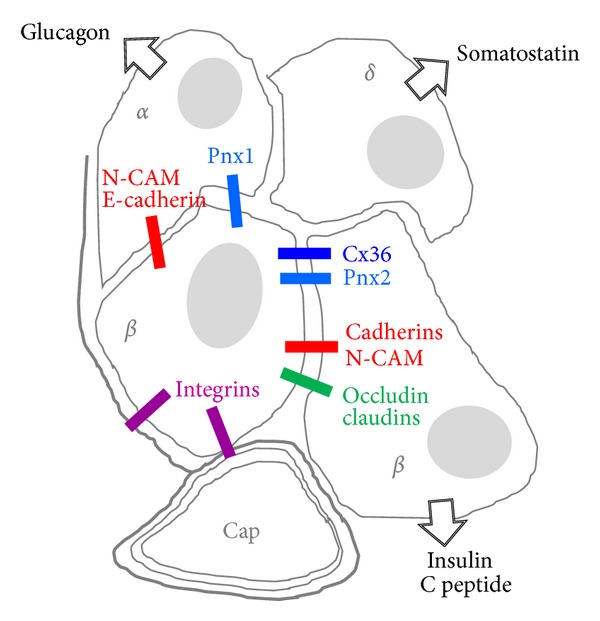
Schematic view of the *β*-cell arrangement within a pancreatic islet. The insulin- and C-peptide-producing *β*-cells interact with each other via Cx36, Pnx2, multiple cadherins, N-CAM, occludin, and several claudin isoforms. *β*-cells also adhere to their basal laminae (grey bands) and to those of the endothelial cells of islet capillaries (cap) by a variety of integrins. *β*-cells further interact with nearby glucagon-producing *α*-cells via Pnx1, and N-CAM. Whether a similar heterocellular interaction takes place with the somatostatin-producing *δ*-cells is not established. Similarly, there is no direct evidence for an interaction mediated by integral membrane proteins between *β*-cells and either the islet ghrelin-producing *ε*-cells or the pancreatic polypeptide-prodcuing PP cells.

Adult islets of control human pancreas express *β*1, *α*V, and *α*3 integrin subunits [101–103] (Figure 3), and the same proteins are also found, together with *α*5 and *α*6, in the islets of several other species [104–108]. However, while the *α*3 subunit is restricted to islet cells, the *α*5 subunit was expressed by both islet and acinar cells [104, 105]. Immunofluorescence revealed that the expression of *α*6/*β*1 integrin varied among rat beta cells, in a way which correlated with the spreading of these cells on the laminin-rich 804G matrix. This spreading was decreased by antibodies against the *α*6/*β*1 integrin or its *β*1 subunit, as well as by antibodies against laminin-332, one of the ligands of *α*6/*β*1 [106, 108]. Within rodent islets, laminin-332, together with laminin-511, type IV collagen, and numerous other laminin chains, including *α*4, *α*5, *β*1, *β*2, and *γ*1, form basal laminae along both islet cells and blood vessels [106, 108]. In human islets, basal laminae are splitted in 2 parts, with some laminin chains facing only endothelial cells and others facing only islet cells [108, 109]. The latter basal laminae are unusual in that they are rich in lutheran glycoprotein, which functions as a laminin *α* chain receptor [109]. The data indicate that multiple receptors, recognizing many components of the ECM, ensure the adhesion and spreading of beta cells to the extracellular materials of pancreatic islets. They further document substantial differences in the composition and arrangement of basal laminae in rodent and human islets.

Insulin secretion is improved when human islet cells are cultured on a ECM made by bovine corneal endothelial cells or of type IV collagen [109, 110], rather than on standard culture plastic. This effect was associated with a decreased transcription of the insulin gene and was dependent on the activation of the ERK pathway [106, 110]. Glucose-induced insulin secretion from rat islets is also stimulated by different ECMs, including endothelial basement membranes, purified fibronectin, and the 804G matrix [111–113], in a way that correlates with the degree of cell spreading, as a result of an upregulation in the expression of *α*6/*β*1 integrin. Thus, secretagogues elicited a higher insulin output from flattened than from spherical beta cells [106]. Comparable observations were made in cultures of canine islet cells, whose expression of *α*3, *α*5, and *α*V decreased with time, coincident with a decrease in proinsulin gene expression, islet insulin content, and stimulated insulin release [105]. Exposure of beta cells to antibodies blocking either the *β*1 integrin subunit or its laminin-332 ligand resulted in a reduction in glucose-stimulated insulin secretion when cells were attached to 804G matrix [108]. The former antibody also inhibited the phosphorylations mediated by FAK, indicating that the out-in signaling activated by the engagement of *β*1 integrins by laminin-332 is relevant to normal beta cell function [108]. These observations demonstrate that the islet cell-matrix interactions, mediated by specific integrins and their cognate glycoprotein ligands, modulate the sensitivity of beta cells to glucose.

The basal laminae (Figure 3) of fetal pancreatic ducts contain laminin-1, fibronectin, and collagen IV [106], and the epithelial cell clusters which bud off these ducts to begin islet morphogenesis express the cognate receptors *α*V/*β*3 and *α*V/*β*5 integrins [106]. Fetal beta-cells also express *α*1/*β*1, an integrin which is strongly induced after islet isolation and culture, and which mediates the migration of fetal beta cells on type IV collagen [106]. Parallel *in vitro* experiments showed that mesenchymal stem cells from umbilical cord blood can be induced to differentiate into pancreatic endocrine cells, by a mechanism which is significantly activated in the presence of ECM, and lead to the formation of three dimensional pseudoislet structures [106, 114, 115]. Proliferation of rodent islet cells is enhanced on a matrix derived from bovine corneal endothelial cells, on a collagen gel and on the 804G matrix [116–120]. While epithelial cells from fetal human pancreas also grow more rapidly on selected ECMs than on standard culture plastic [121], recent data have shown that, after a limited numbers of years, human beta cells are no more able to proliferate *in vitro*, including on the 804G matrix [122], presumably due to epigenetic downregulation of key cell cycle genes. Thus, the effects of integrins and ECM on islet cell growth appear to be cell-, age-, and species-specific. In culture, the survival of isolated islets of Langerhans is prolonged in the presence of some ECM, which probably protects the microorgans from anoikis [123], and isolated beta cells are also partially protected from the apoptosis induced by serum deprivation and by interleukin-1*β*, when cultured on the 804G matrix. While a direct involvement of integrins in the control of beta cell viability has not yet been demonstrated, the expression of *α*3, *α*5, and *α*V integrins at the surface of islet cells decreases with culture time, and this change is coincident with a rise in beta cell apoptosis [105]. The mechanism underlying these effects remains to be fully elucidated. The activity of caspase-8 is reduced in islet cells cultured on ECM, under conditions that increase the activity of focal adhesion kinase (FAK), protein kinase B (PKB, or Akt), and the extracellular signal-regulated kinase (ERK). Conversely, treatment with either an anti-*β*1 integrin antibody, the ERK pathway inhibitor PD98059, or the phosphatidylinositol 3-kinase inhibitor LY294002 increased apoptosis in cells cultured on the 804G matrix, but not on poly-L-lysine [124]. Another study has documented that the 804G matrix induces a transient and moderate increase in the NF-kappaB activity of beta cells [116]. However, this effect is unlikely to account for the improved viability of islet cells, given that the survival of beta cells grown on the 804G matrix was not affected by inhibiting NF-kappaB phosphorylation using either Bay 11-7082, or by preventing this phosphorylation in cells transduced with an adenoviral vector encoding for a nonphosphorylatable form of IkappaB alpha [124]. These experiments indicate that the engagement of integrins with the components of the islet ECM, and notably laminin, may be crucial for islet morphogenesis, as well as for the generation and survival of differentiated beta cells. They also call for direct experiments aimed at elucidating the pathways whereby ECM protects beta cells against apoptosis. 

The migration of autoreactive lymphocytes and other immune cells from the blood stream into pancreatic islets is a pathophysiological culprit in the initiation of type 1 diabetes, which may be controlled by the molecular composition of ECM and the expression of selected CAMs and integrins at the surface of both lymphocytes and endothelial cells [125]. Thus, intercellular adhesion molecule 1 (ICAM-1), which is expressed by beta cells, has also been implicated in the extravasation of lymphocytes from the circulation into the inflamed pancreas [126]. Treatment of nonobese diabetic mice with monoclonal antibodies against L-selectin and the *α*4 subunit of integrins protects against the spontaneous occurrence of insulitis and diabetes [127]. Furthermore, lymphocytes of inflamed islets express the *α*4/*β*7 integrin, and treatment of young diabetic mice with monoclonal antibodies against the *β*7 subunit of this molecule also leads to a significant and long-standing protection against the spontaneous development of diabetes and insulitis [128]. 

In most transplantation centers, isolated pancreatic islets are cultured 1-2 days prior to transplantation for a variety of safety and logistical raisons, as well as to improve the viability of the islets which may have been compromised by the isolation procedure. Since ECM is a native component of the islet microenvironment which positively affects islet cell function *in vitro*, its effects on the survival of transplanted islets have been investigated. In one study, the natural ECM of small intestine submucosa was shown to improve the secretion of isolated islets cultured for several days [129]. Also, when a polymer scaffold made of lactide and glycolide copolymers as well as collagen IV was used as a platform for islet transplantation, to mimic the 3D organization of ECM, the hyperglycemia of diabetic mice was corrected faster than in mice transplanted only with isolated islets [130]. Similar results were obtained with human islets embedded in a synthetic matrix composed of nanofibers, and transplanted into diabetic immunodeficient mice [131]. Chitosan-based artificial matrices have been recently proposed as an alternative to natural ECM for islet transplantation. In these 3D structures, the isolated islets retain the initial morphol-ogy and adequate insulin release for several weeks of culture [132]. These studies indicate that the design of an adequate and well-defined ECM microenvironment and the *in vitro* reestablishment of the cell-ECM interactions which normally take place within the native pancreas may be instrumental to improve the survival and function of transplanted islets.

*Pannexin Mediated*

 The sequencing of mammalian genomes has revealed pannexins, a family of 3 proteins, which feature a membrane topography analogous to that of innexins and connexins [131, 132] (Figure 1). Thus, pannexins display N- and C-terminal domains within the cytoplasm, two extracellular and one cytoplasmic loop domains, and four membrane-spanning segments. However, and in contrast to connexins, pannexins contain two Cys residues in each extracellular loop, and consensus sequences for glycosylation [133–136]. During their intracellular trafficking, 6 pannexins oligomerize to form an hexamer referred to as a pannexon, which is inserted in the cell membrane. This structure forms the wall of a hydrophilic channel which, when opened, establishes a communication between the cytosol and the extracellular fluid (Figure 2). Through pannexin channels, a variety of cytosolic molecules, including ATP, glutamate, and epoxyeicosatrienoic acid [137–139], may escape the cell and either bind to prurinergic receptors on nearby cells or enter these cells, in a channel-dependent variant of paracrine cell-to-cell communication. At least *in vitro*, the pannexon channels also allow cells to rapidly incorporate molecules present in the extracellular fluid, by a gradient-dependent diffusion mechanism [140–144]. In contrast, and probably because of the glycosylated residues, the pannexons of one cell cannot approximate those of an adjacent cell sufficiently close to establish a cell-to-cell channel [135, 136, 142, 143, 145–147]. Pannexin channels are activated by mechanical stress [148–151], large depolarizations, and activation of purinergic receptors [138, 152–159]. These channels have now been implicated in a number of physiological functions, including gene expression, propagation of calcium waves, vasodilation, taste sensation, and immune response [160]. The proteins have also been implicated in a variety of pathophysiological conditions, including cell death, and tumorigenesis [160–162].

Little is known about the function of pannexins in pancreatic islets, beside the finding of transcripts coding for both Pnx-1 and Pnx-2 (unpublished). While cell purification studies indicate that beta-cell-rich fractions predominantly express the RNA of Pnx2, whereas the nonbeta-cell-rich fractions predominantly express the transcript of Pnx-1 (unpublished; Figure 3), neither the cell distribution nor the levels of the cognate proteins are yet established, mostly because of the modest quality of the existing antibodies. In view of the many similarities between beta cells and neurons, a prediction would be that Pnx-2 is selectively expressed by beta cells. Given that these channels can be permeated by glutamate, a putative beta-to-alpha paracrine signal [163], such channels could operate in coordinating the antagonistic insulin and glucagon secretions. Even though the beta-alpha cell interactions have been usually attributed to paracrine communication, a hormonal signaling cannot alone account for all conditions under which beta and alpha cells function antagonistically [51, 53, 163]. The comparison of wild type and pannexin null [161, 164] mice is expected to provide clues about this possibility. At this point, tracer and ATP release experiments have not yet demonstrated that functional Pnx channels operate in isolated mouse islets [147] a negative finding that should take into account that these channels have a rather small conductance and a quite low open probability [160].

#### 5.3.2. Direct Interactions

In spite of some dynamic and quantitative alterations, glucose-induced insulin release is preserved under *in vitro* conditions which perturb the innervation, blood supply, and flux of extracellular fluid which, *in vivo, *mediate the indirect communications between islet cells, discussed in the previous sections. In contrast, physical separation of the islet cells leads to a rapid loss of this regulation, which is at least partially reversible soon after the reestablishment of cell contacts [6, 7, 51, 57]. Hence, maintenance of regulated secretion is dependent on the preservation of at least some of the contacts beta cells established within native pancreatic islets [6, 7, 51, 57]. Three modalities of the direct cell-to-cell communication with contact permits operate within pancreatic islets.

*Receptor/Ligand Mediated*

By bringing cell membranes in close proximity, contact between beta cells allows for the interaction of surface receptors featured by one cell, with surface ligands carried by an adjacent cell. Thus, invalidation of the gene coding for either the insulin or the Igf1 receptor of beta cells unexpectedly resulted in defective glucose-stimulated insulin secretion and impaired glucose tolerance [80, 165], presumably because the intra-islet signaling provided by insulin in either an autocrine and/or paracrine manner is interrupted. A severe impairment of glucose-induced insulin release is also observed after interference with the EphA- and Fas-dependent pathways of *β*-cells, presumably due to perturbed interaction between one of these two receptors and its cognate ligand [166, 167].

*Cell Adhesion Molecule Mediated*

 Most cell types adhere to each other by a variety of single pass, transmembrane proteins, referred to as cell adhesion molecules (CAMs) [168] (Figure 1). Most CAMs are functionally dependent on extracellular Ca^2+^ and thus are referred to as cadherins. These glycoproteins, which have a molecular mass of about 120 KDa, form a family including E, P, N, and R isoforms [169, 170]. The homophilic interaction between cadherins is initiated through dimerization of two cadherin molecules in the membrane of one cell, followed by the homologous interaction of the extracellular domains of one dimer, with the corresponding moieties assembled by an adjacent cell. In the presence of Ca^2+^, the dimers intermingle as the teeth of a zipper, ensuring a stable and strong cell-to-cell adhesion. The intracellular domains of classical cadherins interact with actin myofilaments via several cytosolic proteins, including *α*-actinin, *β*-catenin, *γ*-catenin, and p120 [171]. Cadherins control cell-to-cell adhesion (Figure 2) and, thus, are central to the establishment and maintenance of multicellular organisms. During prenatal development, these molecules play a role in differentiation and morphogenesis of many tissues. In adults, cadherin interactions allow for maintenance of cell polarity and tissue architecture and regulate a variety of functions, including cell growth, motility, and viability. Cell signaling events involving small GTPases of the Rho family have also been shown to be regulated by cadherin engagement [172, 173] (Figure 2). Other CAMs, such as N-CAM, ensure cell-to-cell adhesion independently of Ca^2+^ [173, 174]. N-CAM belongs to the immunoglobulin superfamily and is expressed as 3 distinct isoforms. Two of these isoforms are transmembrane proteins with either a short (N-CAM140) or long (N-CAM180) cytoplasmic domain. The third isoform (N-CAM120) has no cytoplasmic domain and is anchored by glycophosphatidylinositol to the plasma membrane. All three isoforms are posttranslationally modified by the addition of polysialic acid [171, 175]. This glycosylation is regulated during development, decreasing from the embryonic to the adult age [174], in parallel with decreased strength of cell adhesion. 

**Figure 4:**
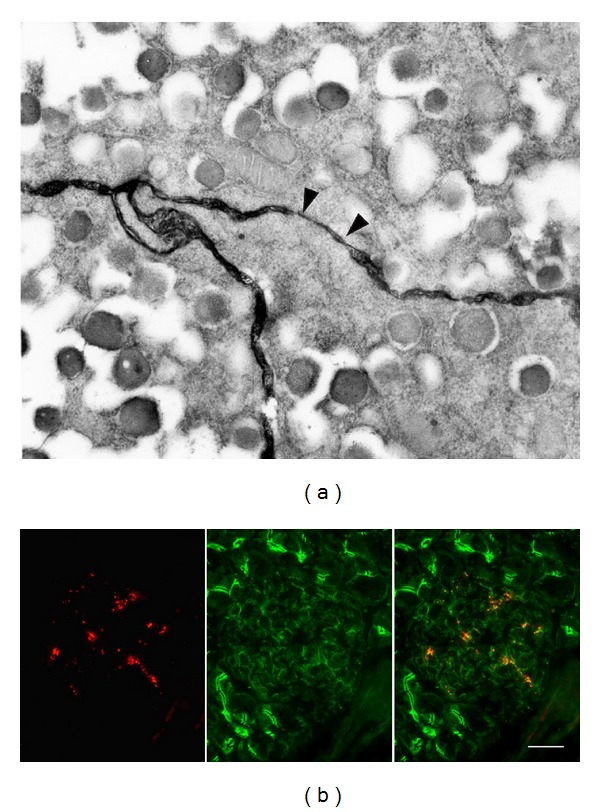
Different integral proteins have specific distributions in the *β*-cell membrane. (a): electron microscopy after en bloc staining of a pancreatic islet with rhutenium red, shows that the extracellular space between adjacent *β*-cells contains abundant glycosylated cell adhesion molecule (black), and is markedly narrowed at the site of a gap junction domain (arrow heads) which concentrated connexons. (b): dual immunofluorescence of a pancreas section reveals the distribution of E-cadherin (green in midlle and right images) along the membrane of *β*-cells in contact, and of exocrine acini. This wide distribution contrasts with the spotted, discrete distribution of Cx36, which is detected only in the small gap junctional domains of *β*-cell membranes (red in left image). The right image is the merge of the left and middle images. Bar: 250 nm in (a), and 50 *μ*m in (b).

Multiple CAMs are expressed in pancreatic islets (Figure 3). N-CAM140 is preferentially found in the non beta cell fraction [168, 174], whereas E-cadherin is the predominant CAM of beta cells [176, 177] (Figure 4). N- and R-cadherins, as well as Ep-CAM have been described in developing, but not adult islets [178–180]. These data show that variable levels of various CAMs, of both Ca^2+^ dependent and independent groups, are expressed by the main endocrine islet cells. This differential expression presumably accounts for the developmental segregation of the hormone-producing cells of pancreas. Thus, both N-CAM and R-cadherin have been detected in the early fetal developmental of the gastroenteropancreatic system, in which they are rapidly segregated into developing islets and related pancreatic ducts, respectively, while being undetectable in the nearby exocrine acini [178, 179]. Later on, R-cadherin decreases in clustered islet cells and becomes undetectable in fully formed islets of Langerhans [181]. Ep-CAM is also thought to have a morphogenetic role in the human pancreas [180]. Thus, high levels of this molecule are found in the growing fetal duct cells which may comprise islet progenitors, as well as in other types of proliferating epithelial cells. In contrast, cells that had started differentiating towards a beta cell phenotype feature low levels of this glycoprotein, which is no more detected in adult human islets [180]. Consistently, antibody blockade of Ep-CAM function promotes the differentiation of fetal human beta cells in culture [180]. 

Rodent pancreatic islets are consistently organized as a core of beta cells surrounded by a peripheral mantle of non beta cells. This specific topographic arrangement is largely dependent on a differential expression of distinct CAMs by beta (E-cadherin > PSA-N-CAM > N-CAM) and non beta cells (E-cadherin > N-CAM). Thus, single islet cells rapidly and spontaneously reaggregate into tri-dimensional organoids, or pseudo-islets, which feature a cellular organization alike that of native islets [182, 183], and this arrangement is perturbed when the reaggregation takes place in the presence of antibodies against N-CAM [184–186]. Over-expression in beta cells of a dominant negative E-cadherin, lacking most of the extracellular domain which is essential for homophilic interaction, abolished the expression of the native cadherin, resulting in defective clustering of beta cells, while alpha cells properly segregated into islet-like structures [187]. Another transgenic mouse, which features diabetes and impaired glucose-stimulated insulin secretion as a result of defective expression of hepatocyte nuclear factors, showed reduced islet expression of E-cadherin [188, 189]. As a result, pancreatic islets also had an altered architecture, with alpha cells scattered throughout both mantle and core of the microorgans [188, 189]. These data strongly support the view that specific CAMs control, at selected developmental stages, the adhesion of endocrine cells into islets and the precise organization of different cell types within these endocrine units. Given, the quite different organization of human pancreatic islets, in which small groups of beta cells appear intermixed with alpha cells and other cell types, which are more abundant than in rodents [190], it remains to be determined to what extent the CAM-dependent sorting also controls the spatial distribution of human islet cells. 

It is well established that insulin secretion from aggregated beta cells is significantly higher than that from an equal number of dispersed cells [72, 73, 76, 78, 191, 192]. Several independent lines of evidence also support the view that beta cells form a functionally heterogeneous population, in terms of insulin biosynthesis and secretion, and that this heterogeneity is compensated within clusters and intact islets, as a result of the establishment of homologous beta cell contacts [6, 51, 57, 76–78]. The sialylated form of N-CAM (PSA-N-CAM) is expressed at different levels in highly and poorly glucose-responsive beta cells [177, 191]. After shedding of PSA using endoneuraminidase N, the recovery of PSA-N-CAM at the beta cell surface was rapidly observed under conditions stimulating insulin secretion, consistent with the localization of the molecule into secretory granules, and its translocation to the cell surface during exocytosis [177]. Interestingly, N-CAM null mice also feature degranulated beta cells, consistent with a role of this CAM in the normal turnover of insulin secretory granules [186]. Insulin secretagogues also promote the expression of E-cadherin in parallel with an increase in the aggregation of rat beta cells and in their glucose-induced insulin secretion [192]. Also, the release of insulin from cultures of MIN6 cells grown in three-dimensional islet-like aggregates was higher than that of the same cells grown in monolayers [193], RNAi-mediated silencing of E-cadherin resulted in a decreased glucose-stimulated secretion, which was reduced to the levels observed in isolated cells [194], and exposure of MIN6 cells to an anti-E-cadherin antibody abolished the glucose-induced increase in cytosolic free calcium [188]. The same antibody also abolished the glucose-stimulated insulin secretion of isolated islets [195]. Still, it is worth noting that the positive effect of E-cadherin on insulin secretion was not confirmed in another study that compared clones of MIN6 cells over- and underexpressing the CAM. These clones did not differ in terms of glucose-induced insulin secretion, even though the levels of preproinsulin mRNA and insulin content, as well as the basal rate of insulin release were higher in over- than in under-expressing cells [196, 197]. These data support the view that CAMs control the postnatal functioning of beta cells, notably by modulating glucose-induced insulin secretion. However, because changes in E-cadherin expression affect the expression of other surface proteins, notably Cx36 [197, 198], the specificity of the CAM-dependent control remains to be established.

Loss of E-cadherin parallels the transition from a well differentiated adenoma to an invasive carcinoma, in a transgenic mouse model of pancreatic beta cell carcinogenesis [199]. In this model, the normal 140-kDa isoform of N-CAM decreases while a 120/180-kDa isoform, which is not expressed in native islets, increases [200]. Under these conditions, the blockade of N-CAM expression favored the development of metastases, suggesting a role of N-CAM in the pathologic dissemination of tumoral beta cells [200]. In pseudo-islets made of transformed MIN6 cells, E-cadherin over expression is associated with increased expression of cyclin-dependent kinase inhibitors, and with a decrease in the cell proliferation marker Ki67, consistent with a role of E-cadherin in some antiproliferation mechanisms [196, 201]. During mouse islet formation, E-cadherin expression increases with the decline in the proliferation rate of beta cells, due to a selective down-regulation of nuclear *β*-catenin and D-cyclins [198]. Together, these data suggest that E-cadherin and N-CAM may contribute to control beta cell proliferation. However, whether such a contribution has any relevance for the minimal, physiological growth of native beta cells remains to be validated, specifically in humans. Intriguingly, the *CDH1* gene, which encodes human E-cadherin, is located on chromosome 16q22.1, a locus that has been implicated in susceptibility to type 1 diabetes [202], and various CAMs have been implicated in the autoimmune pathogenesis of type 1 diabtes [125–128, 203].

CD8^+^ lymphocytes mediate the rejection of pancreatic islet allografts [203]. Some of these cells, referred to as CD103, express the integrin *α*E(CD103)-*β*7, whose only known counterreceptor is E-cadherin [204]. After allotransplantation of control islets, which express E-cadherin, wild type CD103^+/+^ mice rapidly rejected the grafts. In contrast, these grafts indefinitely survived in host CD103^−/−^ mice, which feature a targeted disruption of CD103 [205]. Transfer of CD8^+^ cells into host CD103^−/−^ mice, caused a prompt rejection of the islet allografts, whereas the transfer of CD103^−/−^ and CD8^−/−^ cells had no effect [205]. These results imply that a direct interaction between selected CAMs expressed by both immunocompetent and islet cells may be an important determinant in the acceptance or rejection of a transplanted graft. In turn, this consideration opens the interesting possibility that the engraftment and maintenance of functional islet grafts could be enhanced by a selective modulation of their major CAMs.

*Claudin Mediated*

The 24 members of the claudin family and the 2 isoforms of occluding, which are often associated to claudins, are tetraspan membrane proteins, which feature two extracellular loops, four transmembrane a-helices portions, two cytoplasmic loops, and both N- and C-termini in the cytoplasm [206, 207] (Figure 1). The carboxy terminus of these molecules interacts with different attachment proteins of the ZO, JAM, and cingulin families, each of which comprises multiple isoforms, which functionally interconnect claudins and occludins to the actin microfilaments of the cytoskeleton [207, 208]. Claudins and occludin concentrate at points of focal contact between adjacent cell membranes to form tight junctions. These structures prevent the movement of proteins and lipids between the apical and the basolateral regions of the plasma membrane, thus providing for the structural and functional segregation of these two regions [206, 208] which contributes to cell polarity (Figure 2). By preventing the free diffusion of fluids, solutes and cells across the paracellular space, tight junctions also contribute to impart a selective permeability to groups of epithelial cells. Tight junctions are functionally heterogeneous and form plastic structures whose selective permeability, which varies with their claudin composition, may be modulated by various kinase-dependent mechanisms, triggered by both CAMs and intracellular signals [206, 208]. They further functionally interact with a variety of cytosolic proteins, notably the members of the ZO and JAM families, as well as with tumor suppressor proteins, such as the membrane-associated guanylate kinases [209], indicating that they contribute to cell signaling (Figure 2). 

**Figure 5:**
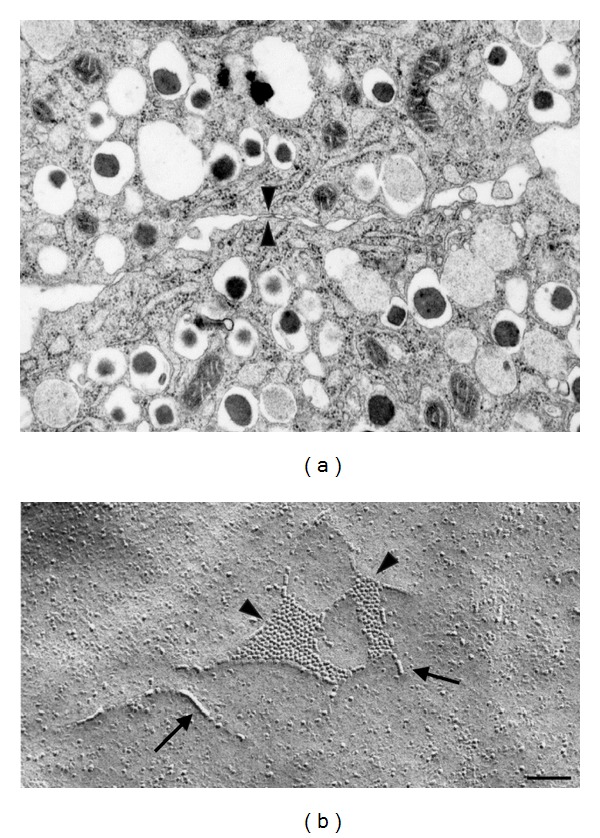
Cx36 mediates the direct coupling of the insulin-producing *β*-cells. (a): transmission electron microscopy shows that the membranes of adjacent *β*-cells, identified by their content in typical insulin-containing granules, are usually separated by an electronlucent extracellular space, which is markedly narrowed at a gap junction plaque (arrow heads). (b): freeze-fracture electron microscopy of such a site reveals clusters of Cx36 channels (arrow heads) within the bilayer of a *β*-cell membrane. The arrows point to short tight junction fibrils, which are closely associated to the connexons. Bar: 250 nm in (a), and 120 nm in (b).

Electron microscopy of pancreatic islets *in situ* has documented sites of contacts between beta cell membranes, which feature all the ultrastructural aspects of *bona fide* tight junctions [210] (Figures 3 and 5). Accordingly, typical tight junctions fibrils have been documented by freeze fracture within the membranes of native beta cells [210, 211]. However, and contrary to what is observed in most other epithelial cells, these fibrils do not form a continuous belt around beta cells and, thus, do not completely seal the intercellular islet space [210–212]. Furthermore, these fibrils are infrequent in islets examined *in situ*, raising concerns about their mere *in vivo* existence [211]. However, both structural and functional evidence exists that islet tight junctions delimit small domains of the beta cell membrane [210, 211, 213], possibly to segregate the portions of the membrane which are rich in hormone receptors and glucose transporters, from those where exocytosis of the insulin-containing secretory granules takes place [79]. Evidence in support of the presence of islet tight junctions also include their rapid *in vitro* modulation [214]. Recently, the expression of some claudins has been reported in genome-wide analysis of islet transcripts, and a major surge of a specific claudin mRNA has been documented in pancreatic islets of pregnant rodents [215, 216]. This unanticipated and striking observation indicates that at least some claudins participate to the islet function *in vivo*. Given that, during pregnancy, beta cells rapidly modify their mass and insulin secretion to adapt to the increased metabolic demand imposed by the foetus growth [217, 218], a careful experimental approach should now test what may be the claudin/tight junction involvement in these structural and functional adaptations.

*Connexin Mediated*

Twenty genes coding for as many connexins (Cx) are found in the human genome, over a large number of chromosomes [6, 35–39]. All connexins feature four membrane-spanning domains linked by two extracellular and one intracellular loop, and cytoplasmic N- and C-terminal regions (Figure 1). The N-terminus, the two extracellular loops, and the four transmembrane domains, which form *α*-helices, have been highly conserved during evolution. In contrast, the intracellular loop and the C-terminus are highly variable [6, 35–39]. Six connexin molecules, of the same or a different type, assemble during their intracellular transport from the ER to the cell membrane, to form the wall of hydrophilic channels [6, 35–39]. After a vesicle-mediated insertion into the cell membrane, these channels concentrate in small domains referred to as gap junctions. At these sites, the intercellular space is reduced to a “gap” of about 2 nm, and the connexons of two adjacent cells join end-to-end, within the extracellular space, to form a junctional channel. Connexin channels are permeable to a variety of ions and larger molecules, including cytosolic metabolites, nucleotides, morphogens, vitamin cofactors, small peptides, and fragments of nucleic acids [1, 4–6]. At gap junctions, the passage of these molecules from one cytoplasm to another is referred to as ionic and metabolic coupling, respectively (Figure 2). The conductance and permeability of both junctional channels and “hemichannels” are highly selective [6, 35–39], as a function of both their Cx composition, and the size, shape, and charge of the permeant molecule [6, 35–39]. Cx channels are open only about 10% of the time and again, depending on their Cx composition, may be gated by transjunctional voltage, cytosol acidification or an increase in free cytosolic Ca^2+^ [1, 4–7]. Gap junction channels have been implicated in the prenatal development, morphogenesis, and differentiation of many tissues, as well as in several functions of adult systems, including cell division and migration, hormonal transmission, electrical and mechanical synchronization, secretion, resistance to cytotoxic agents, compensation of enzymatic defects, and transmission of trophic or deadly molecules [6, 35–39, 57]. The *in vivo* relevance of the Cx functions is supported by the specific phenotypes observed in transgenic mice featuring a knock-in, knockout, or a mutation of selected connexins [6, 35–39, 219, 220]. It is further stressed by the finding of several human diseases which are associated to either Cx mutations or synonymous single nucleotide polymorphisms [6, 35–39, 219, 220]. 

Beta cells are electrically and metabolically coupled by small gap junctions (Figure 4) made of Cx36 [6, 51, 57, 62, 221–230] (Figures 3–5). The electrical coupling encompasses the entire islet, as indicated by the rhythmic and synchronized bursts of electrical activity as well as by the coordinated Ca^2+^ oscillations that are observed during glucose-induced insulin secretion in most beta cells [85, 87, 223, 231–233]. In contrast, the metabolic coupling may be more restricted, since gap junction tracers are only exchanged by small groups of beta cells [59, 227, 229, 230, 234]. Loss of Cx36, after either homologous recombination of the *Gja9* gene or its conditional deletion in beta cells, results in complete beta cell uncoupling and loss of gap junctions, ruling out compensation by another Cx isoform [65, 222, 226, 233, 235, 236]. 

Single beta cells, which can no more be coupled by Cx36 channels, show decreased basal expression of the insulin gene, and reduced proinsulin biosynthesis [236, 237]. Gap junction size correlates with the insulin content of pancreas in rats treated with a sulphonylurea [211, 238]. A correlation has also been documented between the expression of *Gjd2*, the gene which codes for Cx36, and the gene coding for insulin [234, 239, 240]. In the developing mouse pancreas, expression of Cx36 is initially detected at the time the first wave of insulin-producing beta cells is induced [240]. This temporal association is due to the transactivation of *Gjd2* by Beta2/NeuroD1, a transcription factor which is also essential for beta cell differentiation and maturation [241]. Single beta cells also show increased basal release of insulin, and poor to nil glucose-induced insulin release, and elevation in free cytosolic calcium [75, 232, 242–246]. Several of these effects are rapidly reversible after reaggregation [75, 232, 242–246]. Clustering also promotes the recruitment of secretory and biosynthetically active beta cells [77, 237, 243–245]. Comparable conclusions were reached by exposing intact islets to conditions blocking Cx channels [149, 246]. Also, insulin-producing cell lines, which lack normal responsiveness to glucose concentrations, do not express Cx36, whereas lines retaining at least in part the glucose responsiveness of native beta cells do express this connexin [6, 231].

In mice, Cx36 expression increases with the acquisition by beta cells of a normal sensitivity to glucose [239] and is reduced following a high fat diet, which induces glucose intolerance [240]. *In vivo*, transgenic mice whose beta cells overexpress the islet-ectopic Cx32 are also intolerant to glucose, due to decreased glucose-induced insulin release [247]. Cx36 null mice do not release insulin in the normal pulsatile fashion, due to loss of the normal intercellular synchronization of the Ca^2+^ transients induced by glucose stimulation [62, 222, 226, 233]. These islets also show increased basal release of insulin [62, 222, 226, 248], which is a pancreas autonomous defect [222, 231]. The excessive basal secretion is consistent with the finding that uncoupled beta cells can no more be inhibited by hyperpolarizing currents generated in nearby, resting cells [226, 248], and with the prolonged decay in the off response of beta cells once the glucose stimulation stops [226]. The loss of glucose responsiveness is consistent with that of the regular oscillatory output of insulin during both the first and the second phases of insulin release [62, 222, 226, 248]. In mice, loss of Cx36 does not cause overt diabetes [62, 222, 233], but causes intolerance to post-prandial glucose levels as a result of the *in vivo* decrease in the oscillations of circulating insulin [233]. This decrease is associated with alterations in the amplitude and decay [226] of the first phase of glucose-induced insulin secretion, as well as with reduced insulin oscillations during the second phase [233]. The data indicate that Cx36 occupies a prominent hierarchical position in the multifactorial regulation of insulin dynamics, which is central to glycemic control [2, 249]. 

*In vivo*, loss of Cx36 sensitizes beta cells to pharmacological and immunological insults, including by the cytokines which induce apoptosis at the onset of type 1 diabetes [235]. Conversely, transgenic mice overexpressing Cx36, or other Cx isoforms, appear fully protected against the same insults [235]. The mechanism of this protection, which may be partially explained by the extent of beta cell coupling, remains to be fully elucidated. Cx36 may also contribute to the regulation of the beta cell mass, given that apoptosis is a major determinant of beta cell life, and that beta cell coupling is enhanced by hormones prevailing during pregnancy [250], a condition associated with a marked increase in beta cell proliferation and reduced apoptosis. This effect, however, is neither direct, nor linear [62, 222, 251]. 

Cx channels permit a rapid, diffusion-driven, and bidirectional exchange of molecules between coupled cells, a mechanism that rapidly results in the equilibration of mass and electrical gradients [6, 51]. Thus, compared to other mechanisms of cell-to-cell communication, Cx coupling is advantageous in systems in which cell heterogeneity could conceivably result in the asynchronous function of individual cells [6, 51]. Beta cells differ in a number of structural, biochemical, and functional respects, including in terms of the biosynthesis, storage, and release of insulin [4, 6, 57, 77, 219, 228, 232, 242–244, 249–257]. Under such conditions, coupling allows for the recruitment of increasing numbers of secretory cells with both cell aggregation and the degree of stimulation [77, 232, 242–244, 249–254], presumably by counterbalancing the asynchronous function of intrinsically heterogeneous beta cells. In the absence of coupling, such an asynchrony would most likely impair the timely production and release of sufficient amounts of insulin to maintain normoglycemia. The lack of detectable phenotype in heterozygous mice of the Cx36 null and the beta cell Cx36 depleted strains indicates that normoglycemia can be preserved with half the native levels of Cx36 [62, 222, 233, 235]. Estimations based on the minimal conductance of Cx36 channels, required to preserve the intercellular synchronization of the glucose-induced Ca^2+^ transients, indicate that Cx36 signaling is impaired when about 70% of the native Cx36 levels are lost [233]. The reason why normal beta cells need to be coupled is most likely due to the functional heterogeneities of these cells, with respect to multiple structural, biochemical, and functional parameters, notably insulin biosynthesis and secretion [4, 6, 57, 219, 228, 255–257]. 

Coupling may also represent a protective mechanism for beta cells, inasmuch as the irregular Ca^2+^ oscillations, which result from loss of Cx36, could conceivably alter the expression of specific beta cell genes, and the resistance of beta cells to apoptosis [235, 256]. These findings support the notion that glucose-induced insulin secretion is critically dependent on the signaling mediated by beta-to-beta cell contacts. The further finding of Cx36 alterations in experiments testing the effects of the E-cadherin- [193, 197, 198] and EphA-dependent pathways [166] further suggests that Cx36 may be a common partner of several signaling mechanisms which operate within the islets, possibly by providing for their cross-talk and/or final, distal effects. 

Cx36 only forms homomeric and homotypic intercellular channels [6, 35–39], preventing coupled beta cells to share with other cell types the cytosolic signals they need to exchange for coordinating their own activity. Such selectivity is appropriate for proper islet functioning, particularly since beta and alpha cells function antagonistically under most conditions. Gap junctions have been claimed to connect different islet cell types [258], which are occasionally coupled in culture [259, 260]. However, direct evidence that alpha and beta cells make *bona fide* gap junction plaques has not yet been provided, and tracer studies do not support the occurrence of a large coupling between these cells [261–263]. In addition, while both alpha and beta cells feature Ca^2+^ oscillations which are synchronized with those of companion cells, the synchronization of alpha cell oscillations is asynchronous with that of beta cells, in both rodent and human islets [63, 262, 264], implying lack of simultaneous Cx36-dependent coupling between the two cell types. Still, since most secretory cell types express at least one connexin [6, 57], it is not clear why pancreatic alpha cells would be an exception. The regular oscillations of glucagon secretion [63, 263] rather suggests that alpha cells are also coupled, by a mechanism which remains to be unravelled.

The phenotype of mice largely or totally depleted in Cx36 shows several beta cell alterations which are typical of glycemia disorders, including loss of circulating insulin oscillations, glucose intolerance, increased basal secretion, decreased first and second phase of glucose-induced insulin secretion, and increased beta cell apoptosis [51, 62, 235, 248, 252, 256]. Since human beta cells are also coupled by Cx36 [234], whose encoding gene is located in chromosome 15q14 [265], a locus associated with type 2 diabetes [266], an intriguing possibility is that alterations in Cx36 signaling may be implicated in the loss of beta cell function and mass seen in the human clinic [267]. This is further supported by the finding that the expression of Cx36 is down-regulated after chronic exposure to several circulating molecules which contribute to the pathogenesis of these disorders, including high levels of glucose, fatty acids, oxidized low density lipoproteins, and cytokines [235, 268–270]. If there is yet no human evidence for a pathogenic role of Cx36, this concept is supported by several animal and a couple of genetic studies. Mice developing glucose intolerance, obesity, peripheral insulin resistance, hyperglycemia, hyperinsulinemia, and increased beta cell mass after a high fat diet feature decreased glucose-induced insulin secretion, Cx36 expression, and beta cell coupling [240]. Mice invalidated for *Gjd2* feature alterations in beta cell function which are reminiscent of those that precede the development of overt diabetes in humans (e.g., loss of insulin oscillations) and, later, characterize the disease (e.g., increased basal release of insulin and failure to increase the insulin output in the presence of postprandial concentrations of glucose) [51, 62, 235, 248, 252, 256]. A single nucleotide polymorphism in the coding sequence of *GJD2* has been shown to be pathogenic in a form of epilepsia which shares with type 2 diabetes a complex inheritance pattern [271, 272], indicating that subtle genetic Cx36 changes may be pathogenic in humans. A defect in Cx36 signaling could be further enhanced by alterations of circulating nutrients, notably long-term hyperglycemia, and hyperlipidemia, which negatively affects Cx36 expression [267–270]. In mice, the levels of Cx36 correlate with the *in vivo* resistance of *β*-cells to conditions reproducing the apoptosis observed at the onset of type 1 diabetes [235, 256]. Whether this is due to a Cx36-dependent enhancement of their resistance and/or to improved repairing mechanisms remains to be determined. Consistent with these findings, Th1 cytokines decrease Cx36 expression at the transcription level [256], and altered expression of the Cx36 transcript has been detected in genome-wide scans of type I diabetes models [257, 273]. 

In view of the above, the question arises whether we could take advantage of Cx biology to develop innovative therapeutic approaches to glycemic disorders. Strikingly, a sulphonylurea, which stimulates insulin release from beta cells of type 2 diabetics, also promotes the assembly of Cx36 channels and improves beta coupling [211, 227, 238, 249, 274], opening the search for other innovative molecules targeting Cx36 [274]. This connexin is also likely not to be disposable for the forthcoming implementation of cell therapies using surrogate insulin-producing cells to replace the lost or damaged beta cells. This replacement implies that the transplanted cells become functionally integrated within the host tissue, which presumably will involve the development of appropriate Cx36-dependent cell interactions. The embryonic stem and progenitor cells which have so far been tested in this perspective, with modest results in terms of yield of clinically useful insulin-containing cells, lack Cx36 [275–277]. Recently, the forced expression of this connexin isoform has been reported to foster the differentiation of neurons from progenitor cells [278]. Whether the same might apply to beta cells, which share many common features with neurons, including Cx36 expression, remains to be shown. 

## 6. The Present and the Future

The beta cells collectively function as a multicellular system and, as such, are functionally integrated within pancreatic islets by a variety of mechanisms of indirect and direct cell-to-cell communication. We now know that many of the latter mechanisms involve distinct families of integral membrane proteins, which interact, and in part functionally overlap, to ensure the proper function of pancreatic islets. If compelling *in vivo* evidence now supports a physiologically relevant role of at least some of these proteins, many questions remains to be addressed, including what is the molecular and cellular mechanism whereby they control beta and alpha cell functions? What is their hierarchical position in the intricate network of pathways that support the vital insulin and glucagon production? Are these proteins causally implicated in glycemia disorders, and specifically in type 1 and type 2 diabetes? Are they altered in expression and/or functioning as a consequence of these diseases? Could we take benefit of these proteins to develop innovative therapeutic approaches to beta cell diseases? Many challenges and hurdles will undoubtedly complicate the experimental approaches to these questions, particularly *in vivo* and in humans. Still, the challenge is worthwhile, since answering some of these questions is likely to provide novel views on how the pancreatic beta cells interact to ensure proper control of blood glucose and metabolism. In an optimistic perspective, we expect that such a knowledge would set the basis for molecular and cellular targeted treatments of the related disorders, which have an extremely high medical, social and economic cost and have now reached epidemic proportions worldwide. 

## References

[1] LeRoith D, Delahunty G, Wilson GL (1986). Evolutionary aspects of the endocrine and nervous systems. *Recent Progress in Hormone Research*.

[2] Bergsten P, Hellman B (1993). Glucose-induced amplitude regulation of pulsatile insulin secretion from individual pancreatic islets. *Diabetes*.

[3] Gilon P, Ravier MA, Jonas JC, Henquin JC (2002). Control mechanisms of the oscillations of insulin secretion in vitro and in vivo. *Diabetes*.

[4] Tengholm A, Gylfe E (2009). Oscillatory control of insulin secretion. *Molecular and Cellular Endocrinology*.

[5] Henquin JC (2009). Regulation of insulin secretion: a matter of phase control and amplitude modulation. *Diabetologia*.

[6] Bosco D, Haefliger JA, Meda P (2011). Connexins: key mediators of endocrine function. *Physiological Reviews*.

[7] Meda P, Zahnd GR, Wollheim CB (1997). Intercellular communication and insulin secretion. *Contributions of Physiology to the Understanding of Diabeteseds*.

[8] Trinkaus JP (1984). *Cells into Organs. The Forces That Shape the Embryo*.

[9] Edelman GM, Thiery JP (1985). *The Cell in Contact. Adhesions and Junctions as Morphogenetic Determinants*.

[10] Henderson JR (1969). Why are the islets of Langerhans?. *The Lancet*.

[11] Schopf JW (1993). Microfossils of the early Archean Apex Chert: new evidence of the antiquity of life. *Science*.

[12] Bonner JT (2001). *First Signals: The Evolution of Multicellular Development*.

[13] Bonner JT (1998). A way of following individual cells in the migrating slugs of *Dictyostelium discoideum*. *Proceedings of the National Academy of Sciences of the United States of America*.

[14] Bonner JT (1999). The origins of multicellularity. *Integrative Biology*.

[15] Gerhart J, Kirschner M (1997). *Cells, Embryos and Evolution*.

[16] Gerhart J, Kirschner M (2007). The theory of facilitated variation. *Proceedings of the National Academy of Sciences of the United States of America*.

[17] Gerhart J, Lowe C, Kirschner M (2005). Hemichordates and the origin of chordates. *Current Opinion in Genetics and Development*.

[18] Rokas A (2008). The molecular origins of multicellular transitions. *Current Opinion in Genetics and Development*.

[19] Rokas A (2008). The origins of multicellularity and the early history of the genetic toolkit for animal development. *Annual Review of Genetics*.

[20] Wolpert L, Szathmáry E (2002). Multicellularity: evolution and the egg. *Nature*.

[21] Bonner JT (2006). *Why Size Matters: From Bacteria to Blue Whales*.

[22] Buss LW (1999). Slime molds, ascidians, and the utility of evolutionary theory. *Proceedings of the National Academy of Sciences of the United States of America*.

[23] Bull L (1999). On the evolution of multicellularity and eusociality. *Artificial Life*.

[24] Michod RE, Roze D (2001). Cooperation and conflict in the evolution of multicellularity. *Heredity*.

[25] Bern HA (1962). The properties of neurosecretory cells. *General and Comparative Endocrinology*.

[26] Campbell RK, Satoh N, Degnan BM (2004). Piecing together evolution of the vertebrate endocrine system. *Trends in Genetics*.

[27] Gorbman A, Dickoff WD, Vigna SR, Clark NB, Ralph CL (1983). *Comparative Endocrinology*.

[28] LeRoith D, Shiloach J, Roth J, Lesniak MA (1980). Evolutionary origins of vertebrate hormones: substances similar to mammalian insulins are native to unicellular eukaryotes. *Proceedings of the National Academy of Sciences of the United States of America*.

[29] LeRoith D, Roberts C, Lesniak MA, Roth J (1986). Receptors for intercellular messenger molecules in microbes: similarities to vertebrate receptors and possible implications for diseases in man. *Experientia*.

[30] Roth J, Leroith D, Collier ES (1986). The evolutionary origins of intercellular communication and the Maginot lines of the mind. *Annals of the New York Academy of Sciences*.

[31] Roth J, LeRoith D, Collier ES (1985). Evolutionary origins of neuropeptides, hormones, and receptors: possible applications to immunology. *Journal of Immunology*.

[32] Roth J, LeRoith D, Lesniak MA, de Pablo F, Bassas L, Collier E (1986). Molecules of intercellular communication in vertebrates, invertebrates and microbes: do they share common origins?. *Progress in Brain Research*.

[33] Ashcroft FM (2000). *Ion Channels and Disease*.

[34] Bröer S, Wagner CA (2003). *Membrane Transporter Diseases*.

[35] Harris A, Locke D (2008). *Connexin Biology: The Role of Gap Junction in Disease*.

[36] Harris AL (2001). Emerging issues of connexin channels: biophysics fills the gap. *Quarterly Reviews of Biophysics*.

[37] Hervé JC (2005). The connexins, part II. *Biochim Biophys Acta*.

[38] Hervé JC (2004). The connexins, part I. *Biochim Biophys Acta*.

[39] Hervé JC (2005). The connexins, part III. *Biochem Biophys Acta*.

[40] Barrington EJW (1986). The phylogeny of the endocrine system. *Experientia*.

[41] Becker KL, Nylen ES, Snider RH, Becker KL (1990). Endocrinology and the endocrine patient. *Principles and Practice of Emdocrinolgy and Metabolismed*.

[42] Stoka AM (1999). Phylogeny and evolution of chemical communication: an endocrine approach. *Journal of Molecular Endocrinology*.

[43] Blalock JE, Stanton JD (1980). Common pathways of interferon and hormonal action. *Nature*.

[44] Blalock JE (1989). A molecular basis for bidirectional communication between the immune and neuroendocrine systems. *Physiological Reviews*.

[45] Roth J, LeRoith D, Shiloach J (1982). The evolutionary origins of hormones, neurotransmitters, and other extracellular chemical messengers. Implications for mammalian biology. *The New England Journal of Medicine*.

[46] Scharrer B (1978). Peptidergic neurons: facts and trends. *General and Comparative Endocrinology*.

[47] Bern HA (1962). The properties of neurosecretory cells. *General and Comparative Endocrinology*.

[48] Csaba G (1984). The present state in the phylogeny and ontogeny of hormone receptors. *Hormone and Metabolic Research*.

[49] Falkmer S (1993). Phylogeny and ontogeny of the neuroendocrine cells of the gastrointestinal tract. *Endocrinology and Metabolism Clinics of North America*.

[50] van Noorden S, Falkmer S (1980). Gut-islet endocrinology—some evolutionary aspects. *Investigative and Cell Pathology*.

[51] Bavamian S, Klee P, Britan A (2007). Islet-cell-to-cell communication as basis for normal insulin secretion. *Diabetes, Obesity and Metabolism*.

[52] Caton D, Calabrese A, Mas C, Serre-Beinier V, Wonkam A, Meda P (2002). *β*-cell crosstalk: a further dimension in the stimulus-secretion coupling of glucose-induced insulin release. *Diabetes and Metabolism*.

[53] Jain R, Lammert E (2009). Cell-cell interactions in the endocrine pancreas. *Diabetes, Obesity and Metabolism*.

[54] Meda P (1996). The role of gap junction membrane channels in secretion and hormonal action. *Journal of Bioenergetics and Biomembranes*.

[55] Nlend RN, Michon L, Bavamian S (2006). Connexin36 and pancreatic *β*-cell functions. *Archives of Physiology and Biochemistry*.

[56] Meda P, Bosco D, Habener JF, Hussein M (2001). Communication of islet cells: molecules, mechanisms, functions. *Molecular Basis of Endocrine Pancreas Development and Functions*.

[57] Potolicchio I, Cigliola V, Velazquez-Garcia S (2012). Connexin-dependent signaling in neuro-hormonal systems. *Biochimica et Biophysica Acta*.

[58] Brissova M, Powers AC, Seino S, Bell GI (2008). Architecture of pancreatic islets. *Pancreatic Beta Cell in Health and Disease*.

[59] Benninger RKP, Zhang M, Head WS, Satin LS, Piston DW (2008). Gap junction coupling and calcium waves in the pancreatic islet. *Biophysical Journal*.

[60] Gylfe E, Grapengiesser E, Liu YJ, Dryselius S, Tengholm A, Eberhardson M (1998). Generation of glucose-dependent slow oscillations of cytoplasmic Ca^2+^ in individual pancreatic *β* cells. *Diabetes and Metabolism*.

[61] Hellman B, Gylfe E, Grapengiesser E, Lund PE, Berts A (1992). Cytoplasmic Ca^2+^ oscillations in pancreaticfd *β*-cells. *Biochimica et Biophysica Acta*.

[62] Ravier MA, Güldenagel M, Charollais A (2005). Loss of connexin36 channels alters *β*-cell coupling, islet synchronization of glucose-induced Ca^2+^ and insulin oscillations, and basal insulin release. *Diabetes*.

[63] Hellman B, Salehi A, Gylfe E, Dansk H, Grapengiesser E (2009). Glucose generates coincident insulin and somatostatin pulses and antisynchronous glucagon pulses from human pancreatic islets. *Endocrinology*.

[64] Berggren PO, Rorsman P, Efendic S, Flatt PR (1992). Mechanism of action of entero-insular hormones, islet peptides and neural input on the insulin secretory process. *Nutrient Regulation of Insulin Secretion*.

[65] Pipeleers DG, Schuit FC, in't Veld PA (1985). Interplay of nutrients and hormones in the regulation of insulin release. *Endocrinology*.

[66] Stagner JI, Samole E (1991). Pulsatile secretion from the endocrine pancreas : metabolic, hormonal and neural modulation. *The Endocrine Pancreas*.

[67] Wollheim CB, Sharp GW (1981). Regulation of insulin release by calcium. *Physiological Reviews*.

[68] Goodner CJ, Koerker DJ, Stagner JI, Samols E (1991). In vitro pancreatic hormonal pulses are less regular and more frequent than in vivo. *American Journal of Physiology*.

[69] Samols E, Stagner JI, Samols E (1991). Intraislet and islet-acinar portal systems and their significance. *The Endocrine Pancreas*.

[70] Marks V, Samols E, Stagner J, Flatt PR (1992). Intra-islet interactions. *Nutrient Regulation of Insulin Secretion*.

[71] Pipeleers D (1984). Islet cell interactions with pancreatic B-cells. *Experientia*.

[72] Chertow BS, Baranetsky NG, Sivitz WI (1983). Cellular mechanisms of insulin release. Effects of retinoids on rat islet cell-to-cell adhesion, reaggregation, and insulin release. *Diabetes*.

[73] Halban PA, Wollheim CB, Blondel B, Meda P, Niesor EN, Mintz DH (1982). The possible importance of contact between pancreatic islet cells for the control of insulin release. *Endocrinology*.

[74] Maes E, Pipeleers D (1984). Effects of glucose and 3',5'-cyclic adenosine monophosphate upon reaggregation of single pancreatic B-cells. *Endocrinology*.

[75] Lernmark A (1974). The preparation of, and studies on, free cell suspensions from mouse pancreatic islets. *Diabetologia*.

[76] Pipeleers DG (1992). Heterogeneity in pancreatic *β*-cell population. *Diabetes*.

[77] Salomon D, Meda P (1986). Heterogeneity and contact-dependent regulation of hormone secretion by individual B cells. *Experimental Cell Research*.

[78] Bosco D, Orci L, Meda P (1989). Homologous but not heterologous contact increases the insulin secretion of individual pancreatic B-cells. *Experimental Cell Research*.

[79] Kawai K, Ipp E, Orci L (1982). Circulating somatostatin acts on the islets of Langerhans by way of a somatostatin-poor compartment. *Science*.

[80] Kulkarni RN, Brüning JC, Winnay JN, Postic C, Magnuson MA, Kahn CR (1999). Tissue-specific knockout of the insulin receptor in pancreatic *β* cells creates an insulin secretory defect similar to that in type 2 diabetes. *Cell*.

[81] Rorsman P, Trube G (1986). Calcium and delayed potassium currents in mouse pancreatic *β*-cells under voltage-clamp conditions. *The Journal of Physiology*.

[82] Rodriguez-Diaz R, Abdulreda MH, Formoso AL (2011). Innervation patterns of autonomic axons in the human endocrine pancreas. *Cell Metabolism*.

[83] Rodriguez-Diaz R, Dando R, Jacques-Silva MC (2011). Alpha cells secrete acetylcholine as a non-neuronal paracrine signal priming beta cell function in humans. *Nature Medicine*.

[84] Falke LC, Gillis KD, Pressel DM, Misler S (1989). ‘Perforated patch recording’ allows long-term monitoring of metabolite-induced electrical activity and voltage-dependent Ca^2+^ currents in pancreatic islet B cells. *FEBS Letters*.

[85] Meissner HP (1976). Electrophysiological evidence for coupling between *β* cells of pancreatic islets. *Nature*.

[86] Eddlestone GT, Goncalves A, Bangham JA, Rojas E (1984). Electrical coupling between cells in islets of Langerhans from mouse. *Journal of Membrane Biology*.

[87] Meda P, Atwater I, Goncalves A (1984). The topography of electrical synchrony among *β*-cells in the mouse islet of Langerhans. *Quarterly Journal of Experimental Physiology*.

[88] Valdeolmillos M, Nadal A, Soria B, Garcia-Sancho J (1993). Fluorescence digital image analysis of glucose-induced [Ca^2+^]i oscillations in mouse pancreatic islets of Langerhans. *Diabetes*.

[89] Valdeolmillos M, Santos RM, Contreras D, Soria B, Rosario LM (1989). Glucose-induced oscillations of intracellular Ca^2+^ concentration resembling bursting electrical activity in single mouse islets of Langerhans. *FEBS Letters*.

[90] Pérez-Armendariz E, Atwater I, Rojas E (1985). Glucose-induced oscillatory changes in extracellular ionized potassium concentration in mouse islets of Langerhans. *Biophysical Journal*.

[91] Bleich D, Chen S, Gu JL, Nadler JL (1998). The role of 12-lipoxygenase in pancreatic-cells. *International Journal of Molecular Medicine*.

[92] McDaniel ML, Corbett JA, Kwon G, Hill JR (1997). A role for nitric oxide and other inflammatory mediators in cytokine-induced pancreatic *β*-cell dysfunction and destruction. *Advances in Experimental Medicine and Biology*.

[93] Eizirik DL, Flodström M, Karlsen AE, Welsh N (1996). The harmony of the spheres: inducible nitric oxide synthase and related genes in pancreatic beta cells. *Diabetologia*.

[94] Argiles JM, Lopez-Soriano J, Ortiz MA, Pou JM, Lopez-Soriano FJ (1992). Interleukin-1 and *β*-cell function: more than one second messenger?. *Endocrine Reviews*.

[95] Hynes RO (2002). Integrins: bidirectional, allosteric signaling machines. *Cell*.

[96] Giancotti FG, Ruoslahti E (1999). Integrin signaling. *Science*.

[97] Belkin AM, Stepp MA (2000). Integrins as receptors for laminins. *Microscopy Research and Technique*.

[98] Miranti CK, Brugge JS (2002). Sensing the environment: a historical perspective on integrin signal transduction. *Nature Cell Biology*.

[99] Schwartz MA, Ginsberg MH (2002). Networks and crosstalk: integrin signalling spreads. *Nature Cell Biology*.

[100] Parsons JT (2003). Focal adhesion kinase: the first ten years. *Journal of Cell Science*.

[101] Schwartz MA (2001). Integrin signaling revisited. *Trends in Cell Biology*.

[102] Howe AK, Aplin AE, Juliano RL (2002). Anchorage-dependent ERK signaling—mechanisms and consequences. *Current Opinion in Genetics and Development*.

[103] Levine F, Beattie GM, Hayek A (1994). Differential integrin expression facilitates isolation of human fetal pancreatic epithelial cells. *Cell Transplantation*.

[104] Wang RN, Paraskevas S, Rosenberg L (1999). Characterization of integrin expression in islets isolated from hamster, canine, porcine, and human pancreas. *Journal of Histochemistry and Cytochemistry*.

[105] Wang RN, Rosenberg L (1999). Maintenance of beta-cell function and survival following islet isolation requires re-establishment of the islet-matrix relationship. *Journal of Endocrinology*.

[106] Bosco D, Meda P, Halban PA, Rouiller DG (2000). Importance of cell-matrix interactions in rat islet *β*-cell secretion in vitro: role of *α*6*β*1 integrin. *Diabetes*.

[107] Kantengwa S, Baetens D, Sadoul K, Buck CA, Halban PA, Rouiller DG (1997). Identification and characterization of *α*3*β*1 integrin on primary and transformed rat islet cells. *Experimental Cell Research*.

[108] Parnaud G, Hammar E, Rouiller DG, Armanet M, Halban PA, Bosco D (2006). Blockade of *β*1 integrin-laminin-5 interaction affects spreading and insulin secretion of rat *β*-cells attached on extracellular matrix. *Diabetes*.

[109] Kaido T, Yebra M, Cirulli V, Rhodes C, Diaferia G, Montgomery AM (2006). Impact of defined matrix interactions on insulin production by cultured human *β*-cells: effect on insulin content, secretion, and gene transcription. *Diabetes*.

[110] Beattie GM, Lappi DA, Baird A, Hayek A (1991). Functional impact of attachment and purification in the short term culture of human pancreatic islets. *Journal of Clinical Endocrinology and Metabolism*.

[111] Kaiser N, Corcos AP, Tur-Sinai A, Ariav Y, Cerasi E (1988). Monolayer culture of adult rat pancreatic islets on extracellular matrix: long term maintenance of differentiated B-cell function. *Endocrinology*.

[112] Kaiser N, Corcos AP, Sarel I, Cerasi E (1991). Monolayer culture of adult rat pancreatic islets on extracellular matrix: modulation of B-cell function by chronic exposure to high glucose. *Endocrinology*.

[113] Hulinsky I, Harrington J, Cooney S, Silink M (1995). Insulin secretion and DNA synthesis of cultured islets of Langerhans are influenced by the matrix. *Pancreas*.

[114] Jones PM, Courtney ML, Burns CJ, Persaud SJ (2008). Cell-based treatments for diabetes. *Drug Discovery Today*.

[115] Gao F, Wu DQ, Hu YH, Jin GX (2008). Extracellular matrix gel is necessary for in vitro cultivation of insulin producing cells from human umbilical cord blood derived mesenchymal stem cells. *Chinese Medical Journal*.

[116] Hayek A, Lopez AD, Beattie GM (1989). Enhancement of pancreatic islet cell monolayer growth by endothelial cell matrix and insulin. *In Vitro Cellular and Developmental Biology*.

[117] Schuppin GT, Bonner-Weir S, Montana E, Kaiser N, Weir GC (1993). Replication of adult pancreatic-beta cells cultured on bovine corneal endothelial cell extracellular matrix. *In Vitro Cellular and Developmental Biology A*.

[118] Metrakos P, Yuan S, Qi SJ, Duguid WP, Rosenberg L (1994). Collagen gel matrix promotes islet cell proliferation. *Transplantation Proceedings*.

[119] Hayek A, Beattie GM, Cirulli V, Lopez AD, Ricordi C, Rubin JS (1995). Growth factor/matrix-induced proliferation of human adult *β*-cells. *Diabetes*.

[120] Parnaud G, Bosco D, Berney T (2008). Proliferation of sorted human and rat beta cells. *Diabetologia*.

[121] Beattie GM, Rubin JS, Mally MI, Otonkoski T, Hayek A (1996). Regulation of proliferation and differentiation of human fetal pancreatic islet cells by extracellular matrix, hepatocyte growth factor, and cell-cell contact. *Diabetes*.

[122] Lefebvre VH, Otonkoski T, Ustinov J, Huotari MA, Pipeleers DG, Bouwens L (1998). Culture of adult human islet preparations with hepatocyte growth factor and 804 G matrix is mitogenic for duct cells but not for *β*-cells: effect of HGF on human 2-cells. *Diabetes*.

[123] Thomas FT, Contreras JL, Bilbao G, Ricordi C, Curiel D, Thomas JM (1999). Anoikis, extracellular matrix, and apoptosis factors in isolated cell transplantation. *Surgery*.

[124] Hammar E, Parnaud G, Bosco D (2004). Extracellular matrix protects pancreatic *β*-cells against apoptosis: role of short- and long-term signaling pathways. *Diabetes*.

[125] Yang XD, Michie SA, Mebius RE, Tisch R, Weissman I, McDevitt HO (1996). The role of cell adhesion molecules in the development of IDDM: implications for pathogenesis and therapy. *Diabetes*.

[126] Yagi N, Yokono K, Amano K (1995). Expression of intercellular adhesion molecule 1 on pancreatic *β*-cells accelerates *β*-cell destruction by cytotoxic T-cells in murine autoimmune diabetes. *Diabetes*.

[127] Yang XD, Michie SA, Tisch R, Karin N, Steinman L, McDevitt HO (1994). A predominant role of integrin *α*4 in the spontaneous development of autoimmune diabetes in nonobese diabetic mice. *Proceedings of the National Academy of Sciences of the United States of America*.

[128] Yang XD, Sytwu HK, McDevitt HO, Michie SA (1997). Involvement of *β*7 integrin and mucosal addressin cell adhesion molecule-1 (MAdCAM-1) in the development of diabetes in nonobese diabetic mice. *Diabetes*.

[129] Tian XH, Xue WJ, Pang XL, Teng Y, Tian PX, Feng XS (2005). Effect of small intestinal submucosa on islet recovery and function in vitro culture. *Hepatobiliary and Pancreatic Diseases International*.

[130] Salvay DM, Rives CB, Zhang X (2008). Extracellular matrix protein-coated scaffolds promote the reversal of diabetes after extrahepatic islet transplantation. *Transplantation*.

[131] Navarro-Alvarez N, Rivas-Carrillo JD, Soto-Gutierrez A (2008). Reestablishment of microenvironment is necessary to maintain in vitro and in vivo human islet function. *Cell Transplantation*.

[132] Cui W, Kim DH, Imamura M, Hyon SH, Inoue K (2001). Tissue-engineered pancreatic islets: culturing rat islets in the chitosan sponge. *Cell Transplantation*.

[133] Panchin YV (2005). Evolution of gap junction proteins—the pannexin alternative. *Journal of Experimental Biology*.

[134] Shestopalov VI, Panchin Y (2008). Pannexins and gap junction protein diversity. *Cellular and Molecular Life Sciences*.

[135] Boassa D, Ambrosi C, Qiu F, Dahl G, Gaietta G, Sosinsky G (2007). Pannexin1 channels contain a glycosylation site that targets the hexamer to the plasma membrane. *Journal of Biological Chemistry*.

[136] Boassa D, Qiu F, Dahl G, Sosinsky G (2008). Trafficking dynamics of glycosylated pannexin1 proteins. *Cell Communication and Adhesion*.

[137] Jiang H, Zhu AG, Mamczur M, Falck JR, Lerea KM, McGiff JC (2007). Stimulation of rat erythrocyte P2X_7_ receptor induces the release of epoxyeicosatrienoic acids. *British Journal of Pharmacology*.

[138] Kang J, Kang N, Lovatt D (2008). Connexin 43 hemichannels are permeable to ATP. *The Journal of Neuroscience*.

[139] Laird DW (2008). Closing the gap on autosomal dominant connexin-26 and connexin-43 mutants linked to human disease. *Journal of Biological Chemistry*.

[140] Goodenough DA, Paul DL (2003). Beyond the gap: functions of unpaired connexon channels. *Nature Reviews Molecular Cell Biology*.

[141] Sáez JC, Retamal MA, Basilio D, Bukauskas FF, Bennett MVL (2005). Connexin-based gap junction hemichannels: gating mechanisms. *Biochimica et Biophysica Acta*.

[142] Scemes E, Suadicani SO, Dahl G, Spray DC (2007). Connexin and pannexin mediated cell-cell communication. *Neuron Glia Biology*.

[143] Spray DC, Ye ZC, Ramson BR (2006). Functional connexin “hemichannels”: a critical ap-praisal. *Glia*.

[144] Stout C, Goodenough DA, Paul DL (2004). Connexins: functions without junctions. *Current Opinion in Cell Biology*.

[145] Penuela S, Bhalla R, Gong XQ (2007). Pannexin 1 and pannexin 3 are glycoproteins that exhibit many distinct characteristics from the connexin family of gap junction proteins. *Journal of Cell Science*.

[146] Huang Y, Grinspan JB, Abrams CK, Scherer SS (2007). Pannexin1 is expressed by neurons and glia but does not form functional gap junctions. *Glia*.

[147] Scemes E, Bavamian S, Charollais A, Spray DC, Meda P (2008). Lack of “hemichannel” activity in insulin-producing cells. *Cell Communication and Adhesion*.

[148] Bao L, Locovei S, Dahl G (2004). Pannexin membrane channels are mechanosensitive conduits for ATP. *FEBS Letters*.

[149] Bao L, Sachs F, Dahl G (2004). Connexins are mechanosensitive. *American Journal of Physiology*.

[150] Liu HT, Tashmukhamedov BA, Inoue H, Okada Y, Sabirov RZ (2006). Roles of two types of anion channels in glutamate release from mouse astrocytes under ischemic or osmotic stress. *Glia*.

[151] Quist AP, Rhee SK, Lin H, Lal R (2000). Physiological role of gap-junctional hemichannels: extracellular calcium-dependent isosmotic volume regulation. *Journal of Cell Biology*.

[152] Cherian PP, Siller-Jackson AJ, Gu S (2005). Mechanical strain opens connexin 43 hemichannels in osteocytes: a novel mechanism for the release of prostaglandin. *Molecular Biology of the Cell*.

[153] Hickman SE, Semrad CE, Silverstein SC (1996). P2Z purinoceptors. *CIBA Foundation Symposia*.

[154] Locovei S, Scemes E, Qiu F, Spray DC, Dahl G (2007). Pannexin1 is part of the pore forming unit of the P2X_7_ receptor death complex. *FEBS Letters*.

[155] Parpura V, Scemes E, Spray DC (2004). Mechanisms of glutamate release from astrocytes: gap junction “hemichannels”, purinergic receptors and exocytotic release. *Neurochemistry International*.

[156] Pelegrin P, Surprenant A (2006). Pannexin-1 mediates large pore formation and interleukin-1*β* release by the ATP-gated P2X_7_ receptor. *The EMBO Journal*.

[157] Suadicani SO, Brosnan CF, Scemes E (2006). P2X_7_ receptors mediate ATP release and amplification of astrocytic intercellular Ca^2+^ signaling. *The Journal of Neuroscience*.

[158] Suadicani SO, Flores CE, Urban-Maldonado M, Beelitz M, Scemes E (2004). Gap junction channels coordinate the propagation of intercellular Ca^2+^ signals generated by P2Y receptor activation. *Glia*.

[159] Wang ECY, Lee JM, Ruiz WG (2005). ATP and purinergic receptor-dependent membrane traffic in bladder umbrella cells. *The Journal of Clinical Investigation*.

[160] Scemes E, Spray DC, Meda P (2009). Connexins, pannexins, innexins: novel roles of ‘hemi-channels’. *Pflugers Archiv European Journal of Physiology*.

[161] Santiago MF, Veliskova J, Patel NK (2011). Targeting pannexin1 improves seizure outcome. *PLoS ONE*.

[162] Orellana JA, Shoji KF, Abudara V (2011). Amyloid *β*-induced death in neurons involves glial and neuronal hemichannels. *The Journal of Neuroscience*.

[163] Ishihara H, Maechler P, Gjinovci A, Herrera PL, Wollheim CB (2003). Islet *β*-cell secretion determines glucagon release from neigbouring *α*-cells. *Nature Cell Biology*.

[164] Penuela S, Gyenis L, Ablack A (2012). Loss of pannexin 1 attenuates melanoma progression by reversion to a melanocytic phenotype. *The Journal of Biological Chemistry*.

[165] Kulkarni RN, Holzenberger M, Shih DQ (2002). _*β*_-cell-specific deletion of the Igf1 receptor leads to hyperinsulinemia and glucose intolerance but does not alter _*β*_-cell mass. *Nature Genetics*.

[166] Konstantinova I, Nikolova G, Ohara-Imaizumi M (2007). EphA-ephrin-A mediated beta cell communication regulates insulin secretion from pancreatic islets. *Cell*.

[167] Schumann DM, Maedler K, Franklin I (2007). The Fas pathway is involved in pancreatic *β* cell secretory function. *Proceedings of the National Academy of Sciences of the United States of America*.

[168] Takeichi M (1988). The cadherins: cell-cell adhesion molecules controlling animal morphogenesis. *Development*.

[169] Takeichi M (1990). Cadherins: a molecular family important in selective cell-cell adhesion. *Annual Review of Biochemistry*.

[170] Hatta K, Takeichi M (1986). Expression of N-cadherin adhesion molecules associated with early morphogenetic events in chick development. *Nature*.

[171] Gottardi CJ, Gumbiner BM (2001). Adhesion signaling: how *β*-catenin interacts with its partners. *Current Biology*.

[172] Nelson WJ (2008). Regulation of cell-cell adhesion by the cadherin-catenin complex. *Biochemical Society Transactions*.

[173] Chuong CM, Edelman GM (1984). Alterations in neural cell adhesion molecules during development of different regions of the nervous system. *The Journal of Neuroscience*.

[174] Langley OK, Aletsee-Ufrecht MC, Grant NJ, Gratzl M (1989). Expression of the neural cell adhesion molecule NCAM in endocrine cells. *Journal of Histochemistry and Cytochemistry*.

[175] Chuong CM, Edelman GM (1984). Alterations in neural cell adhesion molecules during development of different regions of the nervous system. *The Journal of Neuroscience*.

[176] Rouiller DG, Cirulli V, Halban PA (1991). Uvomorulin mediates calcium-dependent aggregation of islet cells, whereas calcium-independent cell adhesion molecules distinguish between islet cell types. *Developmental Biology*.

[177] Kiss JZ, Wang C, Olive S (1994). Activity-dependent mobilization of the adhesion molecule polysialic NCAM to the cell surface of neurons and endocrine cells. *The EMBO Journal*.

[178] Gaidar YA, Lepekhin EA, Sheichetova GA, Witt M (1998). Distribution of N-cadherin and NCAM in neurons and endocrine cells of the human embryonic and fetal gastroenteropancreatic system. *Acta Histochemica*.

[179] Hutton JC, Christofori G, Chi WY (1993). Molecular cloning of mouse pancreatic islet R-cadherin: differential expression in endocrine and exocrine tissue. *Molecular Endocrinology*.

[180] Cirulli V, Crisa L, Beattie GM (1998). KSA antigen Ep-CAM mediates cell-cell adhesion of pancreatic epithelial cells: morphoregulatory roles in pancreatic islet development. *Journal of Cell Biology*.

[181] Sjodin A, Dahl U, Semb H (1995). Mouse R-cadherin: expression during the organogenesis of pancreas and gastrointestinal tract. *Experimental Cell Research*.

[182] Montesano R, Mouron P, Amherdt M, Orci L (1983). Collagen matrix promotes reorganization of pancreatic endocrine cell monolayers into islet-like organoids. *Journal of Cell Biology*.

[183] Halban PA, Powers SL, George KL, Bonner-Weir S (1987). Spontaneous reassociation of dispersed adult rat pancreatic islet cells into aggregates with three-dimensional architecture typical of native islets. *Diabetes*.

[184] Rouiller DG, Cirulli V, Halban PA (1990). Differences in aggregation properties and levels of the neural cell adhesion molecule (NCAM) between islet cell types. *Experimental Cell Research*.

[185] Cirulli V, Baetens D, Rutishauser U, Halban PA, Orci L, Rouiller DG (1994). Expression of neural cell adhesion molecule (N-CAM) in rat islets and its role in islet cell type segregation. *Journal of Cell Science*.

[186] Esni F, Täljedal IB, Perl AK, Cremer H, Christofori G, Semb H (1999). Neural cell adhesion molecule (N-CAM) is required for cell: type segregation and normal ultrastructure in pancreatic islets. *Journal of Cell Biology*.

[187] Dahl U, Sjödin A, Semb H (1996). Cadherins regulate aggregation of pancreatic *β*-cells in vivo. *Development*.

[188] Yamagata K, Nammo T, Moriwaki M (2002). Overexpression of dominant-negative mutant hepatocyte nuclear factor-1*α* in pancreatic *β*-cells causes abnormal islet architecture with decreased expression of E-cadherin, reduced *β*-cell proliferation, and diabetes. *Diabetes*.

[189] Shih DQ, Heimesaat M, Kuwajima S, Stein R, Wright CVE, Stoffel M (2002). Profound defects in pancreatic *β*-cell function in mice with combined heterozygous mutations in Pdx-1, Hnf-1*α*, and Hnf-3*β*. *Proceedings of the National Academy of Sciences of the United States of America*.

[190] Cabrera O, Berman DM, Kenyon NS, Ricordi C, Berggren PO, Caicedo A (2006). The unique cytoarchitecture of human pancreatic islets has implications for islet cell function. *Proceedings of the National Academy of Sciences of the United States of America*.

[191] Bernard-Kargar C, Kassis N, Berthault MF, Pralong W, Ktorza A (2001). Sialylated form of the neural cell adhesion molecule (NCAM): a new tool for the identification and sorting of *β*-cell subpopulations with different functional activity. *Diabetes*.

[192] Bosco D, Rouiller DG, Halban PA (2007). Differential expression of E-cadherin at the surface of rat *β*-cells as a marker of functional heterogeneity. *Journal of Endocrinology*.

[193] Hauge-Evans AC, Squires PE, Persaud SJ, Jones PM (1999). Pancreatic *β*-cell-to-*β*-cell interactions are required for integrated responses to nutrient stimuli: enhanced Ca^2+^ and insulin secretory responses of MIN6 pseudoislets. *Diabetes*.

[194] Jaques F, Jousset H, Tomas A (2008). Dual effect of cell-cell contact disruption on cytosolic calcium and insulin secretion. *Endocrinology*.

[195] Rogers GJ, Hodgkin MN, Squires PE (1997). E-cadherin and cell adhesion: a role in architecture and function in the pancreatic islet. *Cellular Physiology and Biochemistry*.

[196] Carvell MJ, Marsh PJ, Persaud SJ, Jones PM (2007). E-cadherin interactions regulate *β*-cell proliferation in islet-like structures. *Cellular Physiology and Biochemistry*.

[197] Calabrese A, Caton D, Meda P (2004). Differentiating the effects of Cx36 and E-cadherin for proper insulin secretion of MIN6 cells. *Experimental Cell Research*.

[198] Wakae-Takada N, Xuan S, Watanabe K, Meda P, Leibel RL Molecular basis for regulation of islet *β*-cell mass: the role of E-cadherin.

[199] Peri AK, Wilgenbus P, Dahl U, Semb H, Christofori G (1998). A causal role for E-cadherin in the transition from adenoma to carcinoma. *Nature*.

[200] Perl AK, Dahl U, Wilgenbus P, Cremer H, Semb H, Christofori G (1999). Reduced expression of neural cell adhesion molecule induces metastatic dissemination of pancreatic *β* tumor cells. *Nature Medicine*.

[201] Luther MJ, Davies E, Muller D (2005). Cell-to-cell contact influences proliferative marker expression and apoptosis in MIN6 cells grown in islet-like structures. *American Journal of Physiology*.

[202] Field LL (2002). Genetic linkage and association studies of type I diabetes: challenges and rewards. *Diabetologia*.

[203] Sleater M, Diamond AS, Gill RG (2007). Islet allograft rejection by contact-dependent CD8^+^ T cells: perforin and FasL play alternate but obligatory roles. *American Journal of Transplantation*.

[204] Cepek KL, Shaw SK, Parker CM (1994). Adhesion between epithelial cells and T lymphocytes mediated by E-cadherin and the *α*(E)*β*7 integrin. *Nature*.

[205] Feng Y, Wang D, Yuan R, Parker CM, Farber DL, Hadley GA (2002). CD103 expression is required for destruction of pancreatic islet allografts by CD8^+^ T cells. *Journal of Experimental Medicine*.

[206] Koval M Claudin heterogeneity and control of lung tight junctions.

[207] Cereijido M, Anderon J (2001). *Tight Junctions*.

[208] Steed E, Balda MS, Matter K (2010). Dynamics and functions of tight junctions. *Trends in Cell Biology*.

[209] Kim SK (1995). Tight junctions, membrane-associated guanylate kinases and cell signaling. *Current Opinion in Cell Biology*.

[210] Orci L, Perrelet A, Volk BW, Wellmann KF (1997). Morphology of membrane systems in pancreatic islets. *The Diabetic Pancreas*.

[211] Meda P, Perrelet A, Orci L (1979). Increase of gap junctions between pancreatic B-cells during stimulation of insulin secretion. *Journal of Cell Biology*.

[212] Like AA (1970). The uptake of exogenous peroxidase by the beta cells of the islets of Langerhans. *American Journal of Pathology*.

[213] Kawai K, Ipp E, Orci L (1982). Circulating somatostatin acts on the islets of Langerhans by way of a somatostatin-poor compartment. *Science*.

[214] Orci L, Amherdt M, Henquin JC, Lambert AE, Unger RH, Resold AE (1973). Pronase effect on pancreatic,’ beta cell secretion and morphology. *Science*.

[215] Rieck S, White P, Schug J (2009). The transcriptional response of the islet to pregnancy in mice. *Molecular Endocrinology*.

[216] Schraenen A, de Faudeur G, Thorrez L (2010). MRNA expression analysis of cell cycle genes in islets of pregnant mice. *Diabetologia*.

[217] Genevay M, Pontes H, Meda P (2010). Beta cell adaptation in pregnancy: a major difference between humans and rodents?. *Diabetologia*.

[218] Rieck S, Kaestner KH (2010). Expansion of *β*-cell mass in response to pregnancy. *Trends in Endocrinology and Metabolism*.

[219] Laird DW (2010). The gap junction proteome and its relationship to disease. *Trends in Cell Biology*.

[220] Laird DW (2006). Life cycle of connexins in health and disease. *Biochemical Journal*.

[221] Serre-Beinier V, Le Gurun S, Belluardo N (2000). Cx36 preferentially connects *β*-cells within pancreatic islets. *Diabetes*.

[222] Wellershaus K, Degen J, Deuchars J (2008). A new conditional mouse mutant reveals specific expression and functions of connexin36 in neurons and pancreatic beta-cells. *Experimental Cell Research*.

[223] Mears D, Sheppard NF, Atwater I, Rojas E (1995). Magnitude and modulation of pancreatic *β*-cell gap junction electrical conductance in situ. *Journal of Membrane Biology*.

[224] Meda P, Atwater I, Goncalves A (1984). The topography of electrical synchrony among *β*-cells in the mouse islet of Langerhans. *Quarterly Journal of Experimental Physiology*.

[225] Meissner HP (1976). Electrophysiological evidence for coupling between *β* cells of pancreatic islets. *Nature*.

[226] Speier S, Gjinovci A, Charollais A, Meda P, Rupnik M (2007). Cx36-mediated coupling reduces *β*-cell heterogeneity, confines the stimulating glucose concentration range, and affects insulin release kinetics. *Diabetes*.

[227] Charpantier E, Cancela J, Meda P (2007). Beta cells preferentially exchange cationic molecules via connexin 36 gap junction channels. *Diabetologia*.

[228] Kohen E, Kohen C, Thorell B (1979). Intercellular communication in pancreatic islet monolayer cultures: a microfluorometric study. *Science*.

[229] Meda P, Amherdt M, Perrelet A, Orci L (1981). Metabolic coupling between cultured pancreatic B-cells. *Experimental Cell Research*.

[230] Meda P, Michaels RL, Halban PA (1983). In vivo modulation of gap junctions and dye coupling between B-cells of the intact pancreatic islet. *Diabetes*.

[231] Calabrese A, Zhang M, Serre-Beinier V (2003). Connexin 36 controls synchronization of Ca^2+^ oscillations and insulin secretion in MIN6 cells. *Diabetes*.

[232] Jonkers FC, Jonas JC, Gilon P, Henquin JC (1999). Influence of cell number on the characteristics and synchrony of Ca^2+^ oscillations in clusters of mouse pancreatic islet cells. *The Journal of Physiology*.

[233] Head WS, Orseth ML, Nunemaker CS (2012). Connexin-36 gap junctuiions regulate in vivo first and second phase secretion dynamics and glucose tolerance in the conscious mouse. *Diabetes*.

[234] Serre-Beinier V, Bosco D, Zulianello L (2009). Cx36 makes channels coupling human pancreatic *β*-cells, and correlates with insulin expression. *Human Molecular Genetics*.

[235] Klee P, Allagnat F, Pontes H (2011). Connexins protect mouse pancreatic *β* cells against apoptosis. *The Journal of Clinical Investigation*.

[236] Philippe J, Giordano E, Gjinovci A, Meda P (1992). Cyclic adenosine monophosphate prevents the glucocorticoid-mediated inhibition of insulin gene expression in rodent islet cells. *The Journal of Clinical Investigation*.

[237] Schuit FC, in't Veld PA, Pipeleers DG (1988). Glucose stimulates proinsulin biosynthesis by a dose-dependent recruitment of pancreatic beta cells. *Proceedings of the National Academy of Sciences of the United States of America*.

[238] Meda P, Halban P, Perrelet A (1980). Gap junction development is correlated with insulin content in the pancreatic B cell. *Science*.

[239] Carvalho CPF, Barbosa HCL, Britan A (2010). Beta cell coupling and connexin expression change during the functional maturation of rat pancreatic islets. *Diabetologia*.

[240] Carvalho CP, Oliveira RB, Britan A, Santos-Silva JC (2012). Impaired *β*-cell-*β*-cell coupling mediated by Cx36 gap junctions in prediabetic mice. *American Journal of Physiology*.

[241] Nlend RN, Aït-Lounis A, Allagnat F (2012). Cx36 is a target of Beta2/NeuroD1, which associates with prenatal differentiation of insulin-producing *β* cells. *Journal of Membrane Biology*.

[242] Bosco D, Meda P, Thorens B, Malaisse WJ (1995). Heterogeneous secretion of individual B cells in response to D-glucose and to nonglucidic nutrient secretagogues. *American Journal of Physiology*.

[243] Bosco D, Meda P (1991). Actively synthesizing *β*-cells secrete preferentially after glucose stimulation. *Endocrinology*.

[244] Jonkers FC, Henquin JC (2001). Measurements of cytoplasmic Ca^2+^ in islet cell clusters show that glucose rapidly recruits *β*-cells and gradually increases the individual cell response. *Diabetes*.

[245] Pipeleers D, in't Veld P, Maes E, van De Winkel M (1982). Glucose-induced insulin release depends on functional cooperation between islet cells. *Proceedings of the National Academy of Sciences of the United States of America*.

[246] Meda P, Bosco D, Chanson M (1990). Rapid and reversible secretion changes during uncoupling of rat insulin-producing cells. *The Journal of Clinical Investigation*.

[247] Charollais A, Gjinovci A, Huarte J (2000). Junctional communication of pancreatic *β* cell contributes to the control of insulin secretion and glucose tolerance. *The Journal of Clinical Investigation*.

[248] Benninger RK, Head WS, Zhang M, Satin LS, Piston DW (2011). Gap junctions and other mecha-nisms of cell-cell communication regulate basal insulin secretion in the pancreatic islet. *The Journal of Physiology*.

[249] Meda P (2012). The in vivo *β*-to-*β*-cell chat room: connexin connections matter. *Diabetes*.

[250] Michaels RL, Sorenson RL, Parsons JA, Sheridan JD (1987). Prolactin enhances cell-to-cell communication among *β*-cells in pancreatic islets. *Diabetes*.

[251] Klee P, Lamprianou S, Charollais A (2011). Connexin implication in the control of the murine beta-cell mass. *Pediatric Research*.

[252] Benninger RKP, Remedi MS, Head WS, Ustione A, Piston DW, Nichols CG (2011). Defects in beta cell Ca^2+^ signalling, glucose metabolism and insulin secretion in a murine model of KATP channel-induced neonatal diabetes mellitus. *Diabetologia*.

[253] Stefan Y, Meda P, Neufeld M, Orci L (1987). Stimulation of insulin secretion reveals heterogeneity of pancreatic B cells in vivo. *The Journal of Clinical Investigation*.

[254] Heimberg H, de Vos A, Vandercammen A, van Schaftingen E, Pipeleers D, Schuit F (1993). Heterogeneity in glucose sensitivity among pancreatic *β*-cells is correlated to differences in glucose phosphorylation rather than glucose transport. *The EMBO Journal*.

[255] Haefliger JA, Martin D, Favre D Reduction of connexin36 content by ICER-1 contributes to insulin-secreting cells apoptosis induced by oxidized LDL particles.

[256] Allagnat F, Klee P, Peyrou M (2007). The gap junctional protein connexin36 (Cx36) protects pancreatic beta cells against cytotoxic attacks: a possible role in cytokine-mediated beta cell death. *Diabetologia*.

[257] Kutlu B, Cardozo AK, Darville MI (2003). Discovery of gene networks regulating cytokine-induced dysfunction and apoptosis in insulin-producing INS-1 cells. *Diabetes*.

[258] Orci L, Malaisse Lagae F, Ravazzola M (1975). A morphological basis for intercellular communication between *α* and *β* cells in the endocrine pancreas. *The Journal of Clinical Investigation*.

[259] Meda P, Kohen E, Kohen C (1982). Direct communication of homologous and heterologous endocrine islet cells in culture. *Journal of Cell Biology*.

[260] Ito A, Ichiyanagi N, Ikeda Y (2012). Adhesion molecule CADM1 contributes to gap junctional communication among pan-creatic islet *α*-cells and prevents their excessive secretion of glucagon. *Islets*.

[261] Kanno T, Göpel SO, Rorsman P, Wakui M (2002). Cellular function in multicellular system for hormone-secretion: electrophysiological aspect of studies on *α*-, *β*- and *δ*-cells of the pancreatic islet. *Neuroscience Research*.

[262] Quesada I, Fuentes E, Andreu E, Meda P, Nadal A, Soria B (2003). On-line analysis of gap junctions reveals more efficient electrical than dye coupling between islet cells. *American Journal of Physiology*.

[263] Nadal A, Quesada I, Soria B (1999). Homologous and heterologous asynchronicity between identified *α*-, *β*- and *δ*-cells within intact islets of Langerhans in the mouse. *The Journal of Physiology*.

[264] Nadal A, Quesada I, Soria B (1999). Homologous and heterologous asynchronicity between identified *α*-, *β*- and *δ*-cells within intact islets of Langerhans in the mouse. *The Journal of Physiology*.

[265] Belluardo N, Trovato-Salinaro A, Mudò G, Hurd YL, Condorelli DF (1999). Structure, chromosomal localization, and brain expression of human Cx36 gene. *Journal of Neuroscience Research*.

[266] Mori Y, Otabe S, Dina C (2002). Genome-wide search for type 2 diabetes in Japanese affected sib-pairs confirms susceptibility genes on 3q, 15q, and 20q and identifies two new candidate loci on 7p and 11p. *Diabetes*.

[267] Hamelin R, Allagnat F, Haefliger JA, Meda P (2009). Connexins, diabetes and the metabolic syndrome. *Current Protein and Peptide Science*.

[268] Allagnat F, Martin D, Condorelli DF, Waeber G, Haefliger JA (2005). Glucose represses connexin36 in insulin-secreting cells. *Journal of Cell Science*.

[269] Allagnat F, Alonso F, Martin D, Abderrahmani A, Waeber G, Haefliger JA (2008). ICER-1*γ* overexpression drives palmitate-mediated connexin36 down-regulation in insulin-secreting cells. *Journal of Biological Chemistry*.

[270] Haefliger JA, Martin D, Favre D Reduction of Cconnexin36 content by ICER-1 contributes to insulin-secreting cells apoptosis induced by oxidized LDL particles.

[271] Mas C, Taske N, Deutsch S (2004). Association of the connexin36 gene with juvenile myoclonic epilepsy. *Journal of Medical Genetics*.

[272] Hempelmann A, Heils A, Sander T (2006). Confirmatory evidence for an association of the connexin-36 gene with juvenile myoclonic epilepsy. *Epilepsy Research*.

[273] Rasschaert J, Liu D, Kutlu B (2003). Global profiling of double stranded RNA- and IFN-*γ*-induced genes in rat pancreatic beta cells. *Diabetologia*.

[274] Bavamian S, Pontes H, Cancela J (2012). The intercellular synchronization of Ca^2+^ oscillations evaluates Cx36-dependent coupling. *PLoS ONE*.

[275] Oyamada M, Oyamada Y, Kaneko T, Takamatsu T (2002). Regulation of gap junction protein (connexin) genes and function in differentiating ES cells. *Methods in Molecular Biology*.

[276] Wörsdörfer P, Maxeiner S, Markopoulos C (2008). Connexin expression and functional analy-sis of gap junctional communication in mouse embryonic stem cells. *Stem Cells*.

[277] Bhandari DR, Seo KW, Sun B (2011). The simplest method for in vitro *β*-cell production from human adult stem cells. *Differentiation*.

[278] Hartfield EM, Rinaldi F, Glover CP, Wong LF, Caldwell MA, Uney JB (2011). Connexin 36 expression regulates neuronal differentiation from neural progenitor cells. *PLoS ONE*.

